# Assessing responses of reef island seabirds to environmental and anthropogenic drivers

**DOI:** 10.1111/cobi.70335

**Published:** 2026-07-09

**Authors:** Tristan Berr, Alexandre Millon, Pascal Dumas, Poetea Guehenneuc, Fany Perez, Hélène De Méringo, Julien Baudat‐Franceschi, Éric Vidal

**Affiliations:** ^1^ UMR 250 ENTROPIE, Institut de Recherche pour le Développement Nouméa New Caledonia; ^2^ Aix Marseille Univ, Avignon Univ, CNRS, IRD, IMBE, Technopôle Arbois‐Méditerranée Aix‐en‐Provence France; ^3^ UMR 228 ESPACE‐DEV, Université de la Nouvelle Calédonie Nouméa New Caledonia; ^4^ Univ. Bordeaux Talence France; ^5^ La Rochelle Univ. La Rochelle France; ^6^ Parc Naturel Régional du Luberon Apt France

**Keywords:** biogeography, conservation planning, coral reef crisis, coral reef island, habitat restoration, human disturbance, land–sea interface, seabird conservation, biogeografía, conservación de aves marinas, crisis de arrecifes coralinos, interfaz costera, islas arrecifales, perturbación humana, planeación de la conservación

## Abstract

Tropical insular ecosystems face escalating, cumulative impacts of land‐ and sea‐based ecological threats that jeopardize the long‐term sustainability of their native communities. This joint vulnerability is epitomized by reef island seabirds, which are exposed to reef decline and coastal erosion driven by climate change, reduction in prey availability, human disturbance, and predation by invasive non‐native species. Using site monitoring data acquired from 2000 to 2023, we investigated drivers of species presence and abundance for 15 seabird species (nine Laridae, three Sulidae, two Fregatidae, and one Procellariidae) on 100 coral reef islands around New Caledonia, South Pacific. We established presence and abundance estimates using a compendium of 2805 observations and tested the relative influence of onsite breeding habitat area, characteristics of neighboring marine environments, human visitation, and rodent presence. Responses to ecological drivers varied across species and highlighted distinct functional and taxonomic groups. The presence and abundance of pelagic‐feeding species with high site fidelity were predominantly determined by the availability of breeding habitat, whereas the presence of coastal foragers, including most gulls and terns, appeared constrained by resource availability near breeding sites. Human disturbance had a significant negative influence on the presence of brown noddy (*Anous stolidus*; [−3.41; −1.20]), masked (*Sula dactylatra*; [−3.85; −1.21]), and brown (*Sula leucogaster*; [−4.57; −1.46]) and red‐footed booby (*Sula sula*; [−9.58; −1.20]) and a significant positive influence on the abundance of the commensal silver gull (*Larus novaehollandiae*; [0.03; 0.09]). Our results emphasize the importance of habitat restoration and mitigation of human disturbance for preserving breeding sites of pelagic‐feeding species. However, it may be difficult to sustain populations of coastal seabirds whose presence is mostly driven by small‐scale prey distribution. Thus, a comprehensive approach that fully considers the functional diversity of target seabird communities is needed.

## Abstract (es)

Análisis de las respuestas de las aves de islas arrecifales ante los factores ambientales y antropogénicos

**Resumen:**

Los ecosistemas insulares tropicales se enfrentan a impactos cada vez mayores y acumulativos derivados de amenazas ecológicas terrestres y marinas que ponen en peligro la sostenibilidad a largo plazo de sus comunidades autóctonas. Esta vulnerabilidad conjunta se plasma especialmente en las aves marinas de las islas arrecifales, que se ven expuestas al deterioro de los arrecifes y a la erosión costera provocados por el cambio climático, la reducción de la disponibilidad de presas, las perturbaciones humanas y la depredación por parte de especies invasoras no nativas. Utilizamos datos de seguimiento de los sitios recopilados entre 2000 y 2023, investigamos los factores que determinan la presencia y abundancia de 15 especies de aves marinas (nueve de la familia Laridae, tres Sulidae, dos Fregatidae y una Procellariidae) en 100 islas de arrecifes de coral alrededor de Nueva Caledonia, en el sur del Pacífico. Establecimos estimaciones de presencia y abundancia con un compendio de 2,805 observaciones y evaluamos la influencia relativa de la superficie del hábitat de cría in situ, las características de los entornos marinos vecinos, la visita humana y la presencia de roedores. Las respuestas a los factores ecológicos variaron según la especie y destacaron distintos grupos funcionales y taxonómicos. La presencia y abundancia de especies de alimentación pelágica con alta fidelidad al sitio estuvieron determinadas predominantemente por la disponibilidad de hábitat de cría, mientras que la presencia de aves que se alimentan en la costa, incluyendo la mayoría de gaviotas y charranes, pareció verse limitada por la disponibilidad de recursos cerca de los sitios de cría. La perturbación humana tuvo una influencia negativa significativa en la presencia de *Anous stolidus* (‐3,41; ‐1,20), *Sula dactylatra* (‐3,85; ‐1,21) y *S. leucogaster* (‐4,57; ‐1,46) y *S. sula* (‐9,58; ‐1,20), y una influencia positiva significativa en la abundancia de la gaviota plateada (*Larus novaehollandiae*; [0,03; 0,09]). Nuestros resultados enfatizan la importancia de la restauración del hábitat y la mitigación de la perturbación humana para preservar los lugares de cría de las especies pelágicas. Sin embargo, puede resultar difícil mantener las poblaciones de aves marinas costeras cuya presencia depende principalmente de la distribución de presas a pequeña escala. Por lo tanto, se requiere un enfoque integral que tenga plenamente en cuenta la diversidad funcional de las comunidades de aves marinas en cuestión.

## INTRODUCTION

Conservation at the land–sea interface has gained increased attention in recent years due to developing evidence of the reciprocal benefits of cross‐system environmental management (Álvarez‐Romero et al., 2011; Carlson et al., 2021; Harris et al., 2019). Coral reefs and tropical islands are natural targets for such initiatives due to their combined vulnerability to anthropogenic impacts, such as climate change, overfishing, invasive non‐native species, and artificialization (Fernández‐Palacios et al., 2021; Hughes et al., 2020, 2017), and to the variety of ecological linkages between their marine and terrestrial ecosystems (e.g., nutrient and groundwater fluxes and coastal erosion) (Clay et al., 2020; Gilby et al., 2016; Paytan et al., 2006). The interaction between seabirds and islands and reefs has received increasing attention after guano deposition was shown to contribute to the biogeochemical cycles of terrestrial (Espíndola & Carlo, 2024; Grant et al., 2022) and reef ecosystems (Honig & Mahoney, 2016). Seabird colonies adjacent to coral reefs participate in nitrogen and phosphate transfer at the land–sea interface, which indirectly influences the functioning of reef communities by enhancing nutrient availability and coral growth rates (Graham et al., 2018; Lorrain et al., 2017). Consequently, land‐based management of seabird populations in tropical environments also yields conservation gains in marine ecosystems, which highlights the relevance of management strategies (Benkwitt et al., 2021; Gunn et al., 2023) that consider all aspects of the circular seabird economy (Jones et al., 2025). This is especially true on coral reef islands (CRIs), which are low‐lying, sedimentary structures formed by the accumulation of carbonated materials (coral debris and invertebrate shells) in shallow waters adjacent to reef ecosystems (including atolls and other reef islands adjacent to rocky land masses) (Perry et al., 2011; Stoddart & Steers, 1977). Because CRI accretion relies on reef activity (i.e., growth and maintenance of CRIs require functioning coral reefs [Steibl, Kench, et al., 2024]), there is a positive feedback loop between seabirds, reefs, and CRIs. Seabirds rely on CRIs for breeding while indirectly favoring island sustainability by improving the functioning of neighboring reefs (Benkwitt et al., 2023; Berr, Dias, et al., 2023).

At least 52 seabird species (∼15% of all seabirds) breed on CRIs within the intertropical area. Populations of these species will expectedly face multiple hazards due to future effects of climate change (Berr, Dias, et al., 2023; Steibl, Kench, et al., 2024). As low‐level islands, CRIs are exposed to future sea level rise (SLR), which threatens to accelerate coastal erosion (Pang et al., 2023; Woodroffe, 2008). Although CRIs exhibit some degree of resilience (Aslam & Kench, 2017; Masselink et al., 2020), projected rates of SLR will likely overcome their adaptation potential and cause substantial CRI decline (Kane & Fletcher, 2020). Furthermore, the expected alteration of reef functioning caused by ocean warming and acidification (Kleypas et al., 2021; Pandolfi et al., 2011) will disrupt sedimentary and geomorphological processes that are key to CRI preservation (Cornwall et al., 2021; Perry et al., 2011). Although the extent to which CRIs may disappear over the coming decades is uncertain, their long‐term sustainability is thus of significant concern (Steibl, Kench, et al., 2024). Therefore, one may expect a rarefaction and degradation of breeding sites and breeding habitat available to CRI seabirds, which will make it difficult for managers to effectively conserve their populations and underlying influence on reef ecosystems (Berr, Dias, et al., 2023; Hatfield et al., 2012). This issue extends throughout the entire intertropical area because most CRI‐dependent species are pantropical and occupy large areas worldwide (Berr, Dias, et al., 2023; Steibl, Steiger, et al., 2024).

Past examples of seabird management on CRIs mostly involve invasive rodent eradications, which have benefited seabird colonies (Le Corre et al., 2015; Philippe‐Lesaffre et al., 2023; Russell et al., 2015; Samaniego et al., 2020). However, few studies have documented the biogeography of CRI seabirds or examined the basal determinants of seabird presence. Such approaches are nevertheless key to future seabird management on CRIs because site suitability will limit how species can remain or relocate on a given site under aggravated coastal erosion (Carr, Trevail, et al., 2021; Reynolds et al., 2015). In the face of likely CRI decline, the uninformed selection of target sites for management efforts may negate long‐term effects of localized conservation measures such as species eradication, mitigation of human disturbance, or habitat restoration (Reynolds et al., 2015).

For Cuba and New Caledonia, Berr, Millon, et al. [2023] and Garcia‐Quintas, Roy, et al. [2023], respectively, found that breeding site (i.e., island) selection in CRI seabirds is influenced by a mixture of terrestrial (site shape and area and plant cover) and marine drivers (proportion of adjacent reefs and pelagic waters, sea surface temperature, and chlorophyll‐a concentration). Berr, Millon, et al. (2023) highlight a negative impact of human visitation on the species richness and biomass of seabird communities. In larids, nest‐site selection can be determined by microhabitat patterns (e.g., plant distribution and distance to the shore), which further constrain seabird distribution (Garcia‐Quintas, Roy, et al., 2023). Although seabird restoration initiatives (translocation of breeding colonies, social attraction, and habitat restoration) have gained traction with managers, they are still underused for CRI seabirds. In 2023, only ∼150 of the 852 documented restoration projects at the global scale (https://www.seabirddatabase.org/) had been conducted in the intertropical area, most of which occurred on volcanic islands (Spatz et al., 2023). This relative paucity of conservation evidence for CRI seabirds reinforces the need for prospective assessments of species responses to environmental and anthropogenic drivers to identify relevant conservation targets and orient future management strategies (Berr, Millon, et al., 2023; Carr, Trevail, et al., 2021).

Using New Caledonia, South Pacific (21.2°S, 165.9°E), as a case study, we built on the previous analysis of Berr, Millon, et al. (2023) to evaluate the effects of ecological and anthropogenic drivers on site selection in 15 seabird species that breed on CRIs. We used seabird monitoring data collected on 100 CRIs to assess individual species’ responses (i.e., changes in presence likelihood) to habitat availability (proxy of a site's carrying capacity), typology of neighboring marine environments, human visitation, and invasive rodent presence. We also measured the influence of breeding habitat area and human visitation on abundance (i.e., colony size), including only sites that hosted breeding populations. We then considered the implications of our results for the conservation and restoration of CRI seabird populations in the context of possible future CRI decline.

## METHODS

### Study area

**FIGURE 1:**
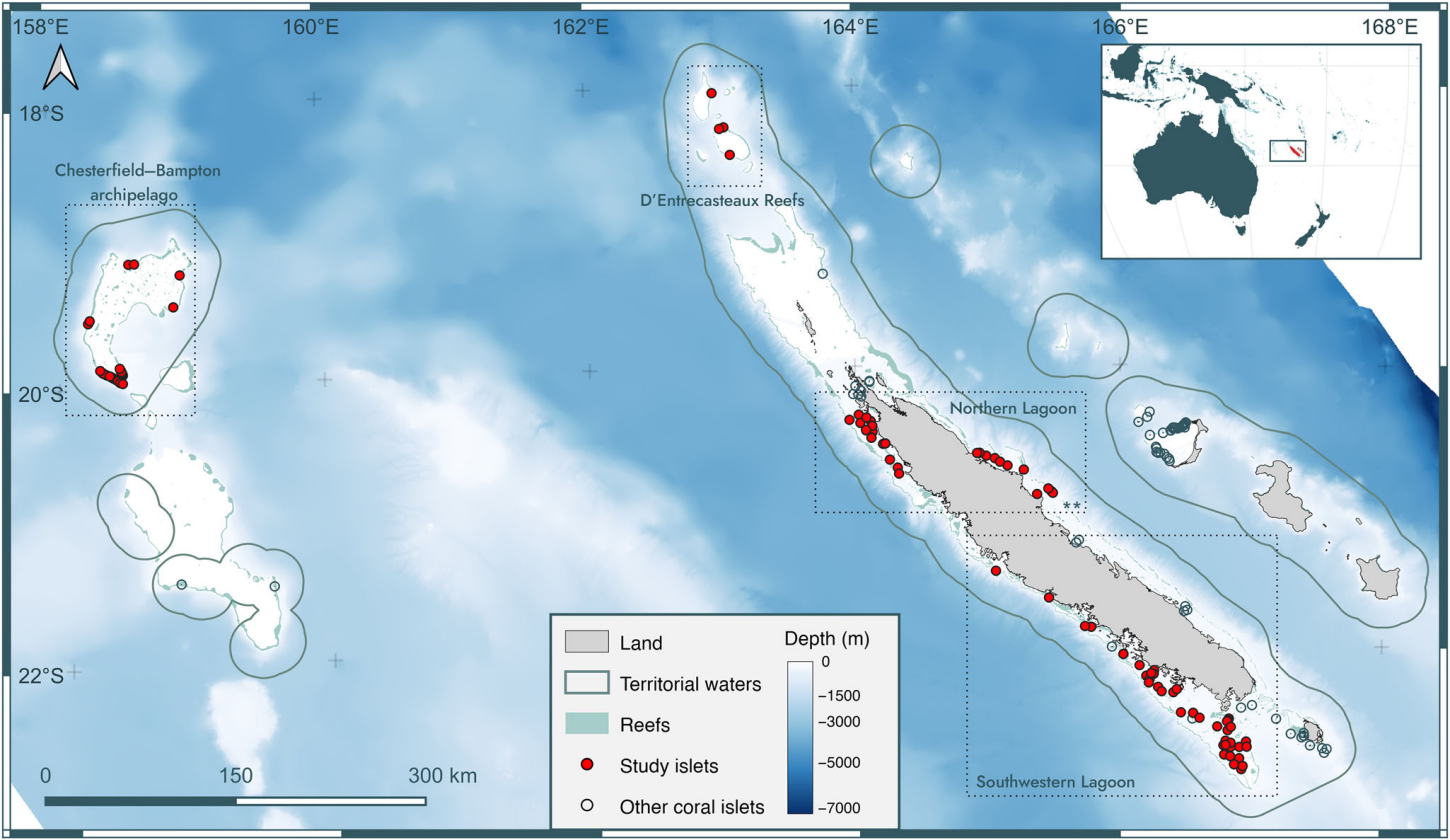
Geographic distribution of study sites in New Caledonia waters used in an examination of drivers of the distribution of breeding seabirds on coral reef islands. Bathymetry and administrative boundaries from Data Source: Government of New Caledonia. Adapted from Berr, Millon, et al. (2023).

The set of CRIs we considered was included in a previous work by Berr, Millon, et al. (2023) and encompasses 100 individual sites (one site = one CRI) from four subregions of New Caledonia (Chesterfield‐Bampton archipelago, 32 CRIs; d'Entrecasteaux reefs, four; northern mainland lagoon, 24; and southwestern lagoon, 39) (Figure 1; Table 1; Appendix S1). New Caledonia is in the southeastern Coral Sea, approximately 1300 km to the east of Australia and 1000 km to the west of Fiji. The archipelago is composed of 700–800 islands, among which ∼160 are CRIs (Data Source: Government of New Caledonia). Its 270,000 inhabitants live predominantly on the mainland and the three Loyalty islands: Ouvéa, Maré, and Lifou. The CRIs from the Chesterfield–Bampton and d'Entrecasteaux subregions (36 sites) (hereafter remote CRIs) are uninhabited; however, they had temporary human settlements in the 19th and early 20th centuries for guano mining and whaling (Borsa & Vidal, 2018; Garrigue et al., 2018). The remaining sites (64) (hereafter mainland CRIs) are in the mainland's lagoon and are close to the coast (<15 km) except for the most southern CRIs that are distributed up to 50 km from the mainland (Figure 1). Among mainland sites, only two CRIs located close to the capital city Nouméa are permanently inhabited due to the presence of tourist infrastructure (∼10 permanent residents each). Platform and barrier islands were present in roughly equal proportions among the study sites.

**TABLE 1:** List and number of breeding sites in the study area for the 15 seabird species in a study of drivers of the distribution of breeding seabirds on coral reef islands.

Family and scientific name		Common name	Foraging range^a^	No. of breeding sites				Percentage of total population on mainland islands
				Remote islands		Mainland islands		
				CB	EN	NL	SWL	
Total				32	4	25	39	
Laridae								
	*Anous minutus*	Black noddy	Offshore	13	2	0	9	51.7
	*Anous stolidus*	Brown noddy	Offshore	20	4	0	14	11.1
	*Larus novaehollandiae*	Silver gull	Neritic	0	0	12	21	100
	*Onychoprion anaethetus*	Bridled tern	Neritic	0	2	3	22	97.2
	*Onychoprion fuscatus*	Sooty tern	Pelagic	9	2	0	4	<0.1
	*Sterna dougallii*	Roseate tern	Neritic	1	0	7	26	99.9
	*Sterna sumatrana*	Black‐naped tern	Neritic	7	2	10	24	67.9
	*Sternula nereis*	Fairy tern	Neritic	3	0	10	8	82.6
	*Thalasseus bergii*	Greater‐crested tern	Offshore	3	3	6	17	82.0
Procellariidae								
	*Ardenna pacifica*	Wedge‐tailed shearwater	Pelagic	8	4	15	32	87.1
Fregatidae								
	*Fregata ariel*	Lesser frigatebird	Pelagic	8	2	0	0	0
	*Fregata minor*	Great frigatebird	Pelagic	12	2	0	0	0
Sulidae								
	*Sula dactylatra*	Masked booby	Pelagic	16	4	1	1	0.3
	*Sula leucogaster*	Brown booby	Pelagic	17	4	1	1	1.7
	*Sula sula*	Red‐footed booby	Pelagic	15	3	0	2	1.2
Number of different species				13	13	9	15	
Species richness (mean ± SD)				4.09 ± 3.55	9.25 ± 0.96	2.6 ± 1.6	5 ± 2.66	

Abbreviations: CB, Chesterfield–Bampton archipelago; EN, d'Entrecasteaux Reef; NL, northern lagoon; SWL, southwestern lagoon.

^a^ Inferred from foraging distance categories summarized by Tobias et al. (2022): <10 km (neritic), <50 km (offshore), and >50 km (pelagic).

Out of the 28 species known to breed in New Caledonia, 18 seabird species breed on at least one CRI within the study area (Berr, Millon, et al., 2023; Spaggiari et al., 2006). Their combined populations amount to >500,000 breeding pairs, of which 93% belong to three species: wedge‐tailed shearwater (*Ardenna pacifica*) (250,000 breeding pairs), sooty tern (*Onychoprion fuscatus*) (140,000), and black noddy (*Anous minutus*) (70,000) (Appendix S2). Three species with fewer than 10 confirmed breeding sites (red‐tailed tropicbird [*Phaethon rubricauda*], three sites; Tahiti petrel [*Pseudobulweria rostrata*], seven; and black‐winged petrel [*Pterodroma nigripennis*], five) were dropped from subsequent analyses because they offered limited statistical power to discriminate drivers of site selection. We considered the remaining 15 species (Table 1). All are classified as least concern by the International Union for Conservation of Nature except the fairy tern (*Sternula nereis*), which is listed as vulnerable (BirdLife International, 2026). We further categorized species by foraging behavior, based on foraging distances compiled by Tobias et al. (2022). Five species were classified as neritic feeders (typical foraging range <10 km), three as offshore (<50 km), and the remaining seven species as pelagic (>50 km).

Local threats to seabirds include human disturbance (visitation of CRIs by tourists, campers, and local fishers), which is mostly concentrated on mainland CRIs (Gonson et al., 2016), and introduced animals and plant species (Caut et al., 2009; Gargominy et al., 1996). Fifteen of the 100 considered CRIs currently host rodent populations, among which six host black rats (*Rattus rattus*), four host house mice (*Mus musculus*), two host Polynesian rats (*Rattus exulans*), and four host unidentified rodent species (Appendix S1). Rodents were originally present at a larger set of sites (30–40 CRIs) but were successfully eradicated in the 1990s and 2000s (Baudat‐Franceschi et al., 2008; Bell, 1998). However, the scarcity of long‐term monitoring data on New Caledonia seabirds has historically impeded the assessment of demographic trends on CRIs from which rodents were eradicated, with Surprise Island (d'Entrecasteaux reefs) being the exception (Philippe‐Lesaffre et al., 2023). Seabirds—especially procellariids—are also exposed to plastic ingestion, although less severely than in neighboring Australia (Berr et al., 2020). Negative impacts of other potential threats, such as light pollution, bycatch, and egg harvest, have not been quantified precisely in New Caledonia, although they are mentioned in gray literature. Finally, for all CRIs, future erosion risks are a growing concern, even if past assessments show differing sedimentary dynamics across monitored sites (Garcin et al., 2016). Because most of the existing information on threats remains qualitative, their relative impacts or local management importance are broadly unknown, which does not allow managers to build hierarchized conservation frameworks.

### Collection and curation of seabird census data

The seabird census data we used were similar to those used by Berr, Millon, et al. (2023). We collected information on seabird presence and abundance from the available literature (including gray literature) published from 2000 to 2023 and conducted new surveys within the four subregions of interest from 2018 to 2023 (Appendix S3). The complete dataset contains 2805 observation entries. We characterized species as present on a CRI if there was at least one confirmed breeding record during the considered period. Species were deemed absent if they were not observed during surveys or if their breeding status was uncertain. For each site, the list of breeding species consequently included species with high site fidelity (e.g., wedge‐tailed shearwater) and species with occasional presence (e.g., fairy tern). We then produced abundance estimates for each species and site by averaging count data across surveys (one value per breeding season during which the species was surveyed). When multiple counts of a colony occurred within the same breeding season, we selected the highest estimate as reference. For species with occasional breeding, years during which the species was absent were included as zero counts to avoid overestimating the overall population size. The resulting estimates of presence and abundances were used as response variables in the subsequent analyses.

To assess dissimilarity between species assemblages across subregions, we produced a visual summary of the community matrix (presence–absence data, 100 sites × 15 species) with nonmetric dimensional scaling (NMDS). We also tested for significant differences with a pairwise analysis of similarities (ANOSIM) between all pairs of subregions.

### Assessment of environmental predictors

Gridded habitat maps (5 × 5 m) had previously been produced for the 100 study CRIs through the interpretation of aerial imagery (drone and satellite photography) (Berr, Millon, et al., 2023). We measured breeding habitat area for each species by assigning preferred habitat categories to each species and summing the area of the corresponding categories to produce an area estimate per species and per CRI. We partly derived habitat categories based on the methodology of Carr, Trevail, et al. (2021), who characterized habitat use by seabird species in the Chagos archipelago, Indian Ocean. In the near absence of wetlands and non‐native forests (coconut groves), we reduced the number of categories from six to five: beach or bare substrate, savanna or low plant cover, mixed shrub (octopus bush tree [*Heliotropium arboreum*] or *Pisonia grandis*), bay cedar (*Suriana maritima*), and other (i.e., a priori unfavorable habitat, mainly composed of undifferentiated forest areas and rarely mangrove). Preferred habitat categories were assigned to each species according to observations by Carr, Trevail, et al. (2021) and local expert opinion (Appendix S4).

To test the influence of a site's surroundings on site selection, we measured the surface of land, lagoon, reefs, and pelagic waters in eight areas of varying radius (0.5–200 km) around each CRI. We then summarized the resulting matrix (100 sites × 32 categories of marine habitats) with principal component analyses (PCAs) and subsequently kept the first two dimensions as predictors in a manner consistent with Berr, Millon, et al. (2023): marine.1, 42.8% of overall variance, and marine.2, 20% of overall variance. This approach was derived from similar work conducted on reef fish communities (D'agata et al., 2014) in the absence of high‐resolution data on ocean productivity and island mass effects in New Caledonia's exclusive economic zone. The underlying assumptions were that reef, lagoon, and pelagic waters offer distinct foraging resources for seabirds (Catry, Ramos, Jaquemet, et al., 2009) and that land acts as a passive obstacle to feeding. Marine.1 could be approximated as a measure of a site's geographic isolation from the mainland because its values strongly differed between mainland and remote CRIs (Appendix S5). Marine.2 was positively correlated to the proportion of lagoon marine habitats up to 50 km from the shoreline and of pelagic waters beyond (Appendix S5).

The level of human disturbance was evaluated by aggregating multiple measures of site accessibility (distance to the closest harbor and to the capital city Nouméa) and exposure to human visitation (number of inhabitants in neighboring cities), as well as categorical descriptions of tourist infrastructure and nature reserve status (Appendix S6). The combined variables were synthetized through multifactorial analyses (MFAs) to reduce the number of predictors and prevent model overfitting. We chose MFA because it provides an extension of PCA that allows the inclusion of categorical variables. The first dimension, hereafter human visitation, captured >50% of the overall variance and was kept as a proxy for disturbance. Its lowest values were associated with the remote CRIs of the Chesterfield archipelago, which are inaccessible to most tourists and whose access is restricted. Conversely, the highest values were found on CRIs closest to the capital city Nouméa, some of which host permanent tourism infrastructure (Appendices S5 & S7).

Finally, information on rodent presence was included as a predictor; however, due to the limited number of sites that had rodent populations (15 out 100), only a binary characterization was used (0, absence of rodents; 1, one or more species among black rat, Polynesian rat, and house mouse).

### Model design and selection

Species‐wise variations in site selection were tested against a set of six predictors: log‐transformed area of breeding habitat, marine.1 and marine.2, visitation, rodent presence, and islet shape (Polsby–Popper score of shape compactness [score close to 1 indicates shape is nearly a circle]). The area of breeding habitat was correlated with total islet surface (Pearson's correlation coefficient >0.7); therefore, the latter was not considered because it was deemed less informative. Marine.1 and visitation were also correlated; thus, they were considered mutually exclusive when comparing models. With the exception of habitat area, all predictors were *z* standardized prior to analyses.

Site selection was modeled with a generalized linear model (GLM) with a logit link and additive effects only to prevent model overfitting (100 sites × 6 predictors). Predictor contributions to the response variable were first evaluated by calculating Akaike weights associated with each predictor rather than performing a standard Akaike information criterion (AIC) comparison of models. Akaike weights measure the proportion of total predictive power associated with each tested model (48 possible combinations), and individual predictor weight can be evaluated as the sum of the weights of all models in which the predictor is retained (Wagenmakers & Farrell, 2004). Predictor weights can then be used to compare predictor influence across species, regardless of effect size, with values close to 1 indicating a strong influence and 0 an absence of measurable effect.

Following the prior evaluation of predictor weights, we produced individual species models for site selection with AIC‐based model averaging (Burnham & Anderson, 2004; Lukacs et al., 2010). Islet shape was discarded from the predictor set because its contribution was not significant in any of the considered species. Due to the correlation between site isolation (marine.1) and human visitation, only the predictor with the highest Akaike weight among the two was retained for model averaging. We observed no spatial autocorrelation in the residuals of the final models (Moran's *I*, *p* > 0.05); *p* values were adjusted for multiple testing, using the Benjamini/Hochberg method to control the false discovery rate (Benjamini & Hochberg, 1995).

We tested the quantitative influence of breeding habitat area on abundance (i.e., colony size) with a Poisson‐link GLM in which only CRIs where species were present were included. Due to the limited number of occupied sites (10–59), only habitat area was used as a continuous predictor to allow for cross‐species comparison. We tested interaction between habitat area and a simplified visitation estimate (below or above the median level of visitation for sites hosting each species), excluding six species only or mostly found on remote CRIs (frigatebirds, boobies, and the sooty tern) that are exposed to very limited visitation. We tested a similar interaction for rodent presence for two species (wedge‐tailed shearwater and silver gull) that had more than three breeding sites with rodents.

Statistical analyses were conducted in R 4.2.2 (R Core Team, 2023) and included the use of the packages FactoMineR (PCA and MFA construction), MuMIn (model averaging and Akaike weight estimation), and spdep (analysis of spatial autocorrelation) (Bartoń, 2023; Bivand et al., 2023; Husson et al., 2023). All GIS analyses were conducted in QGIS 3.16 and 3.28.

## RESULTS

### Species distribution patterns

**FIGURE 2:**
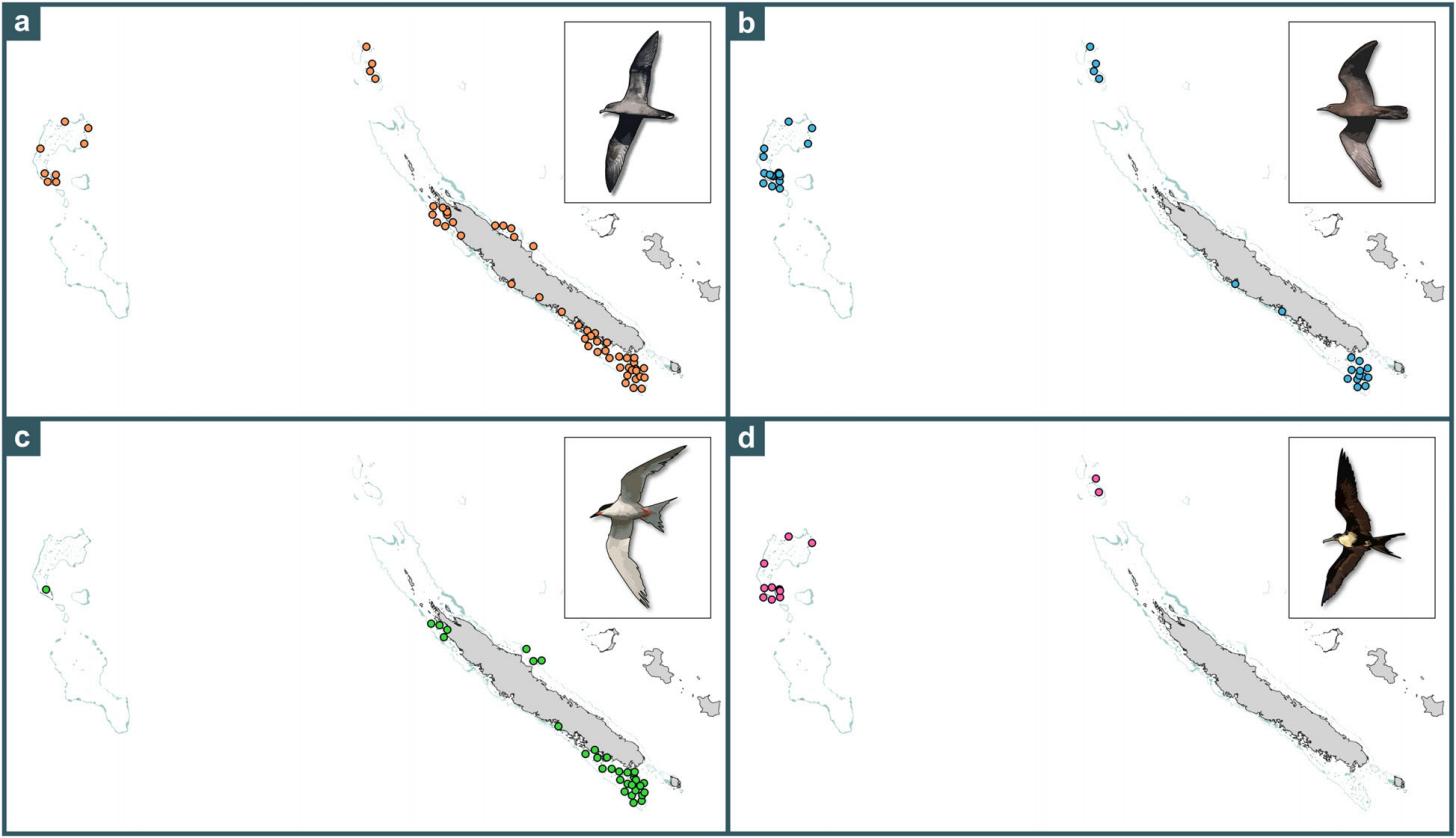
Distribution of breeding sites for (a) wedge‐tailed shearwater, (b) brown noddy, (c) roseate tern, and (d) lesser frigatebird on New Caledonia coral reef islands.

**FIGURE 3:**
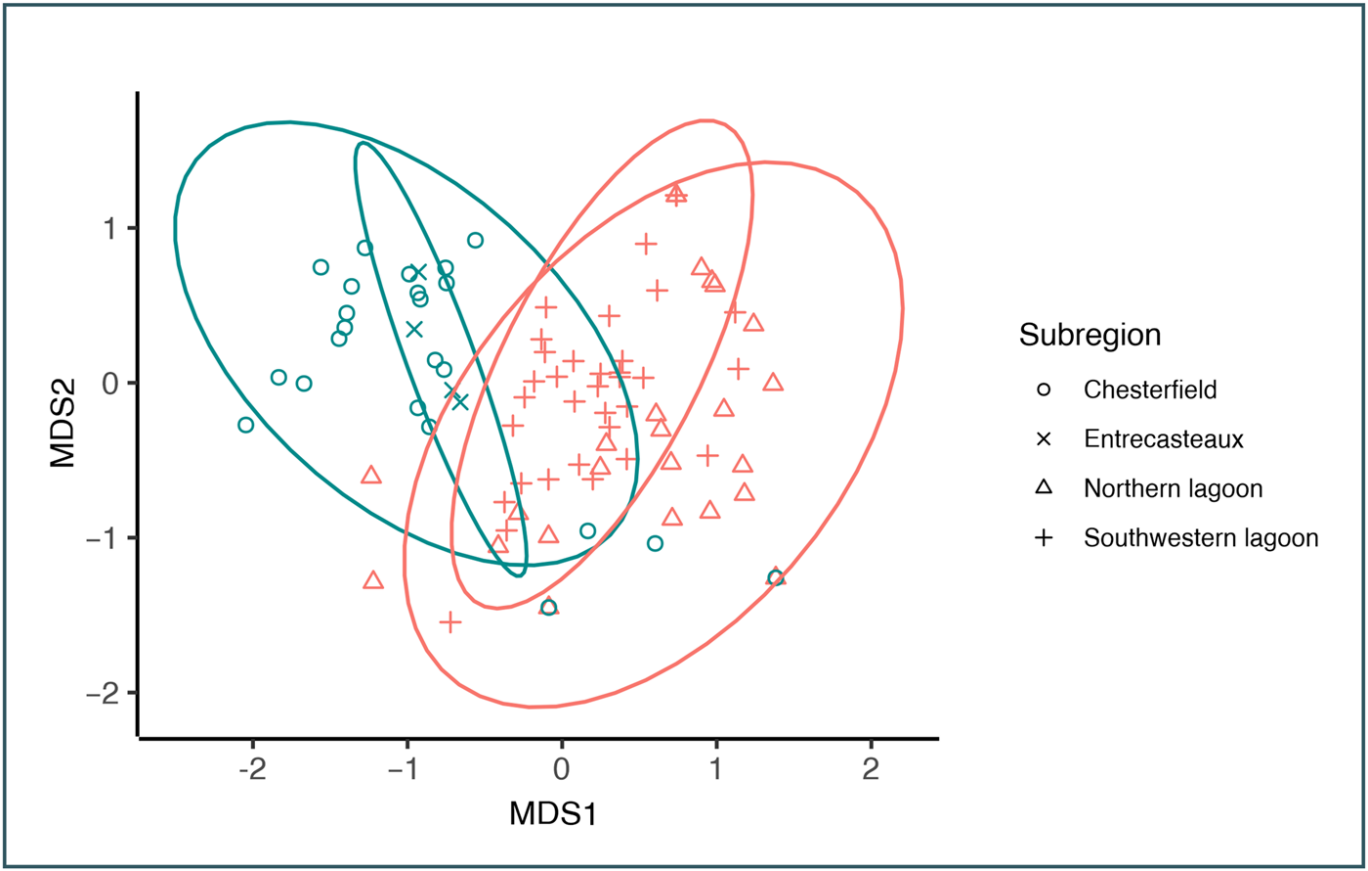
Seabird species assemblages on remote (blue) and mainland (orange) coral reef islands across the four considered subregions obtained through nonmetric dimensional scaling (NMDS) applied to the presence–absence matrix (100 sites × 15 species; stress estimate 0.155).

Species exhibited contrasted distribution patterns within the study area (Figure 2). The wedge‐tailed shearwater was the most widely distributed species (total of 59 breeding sites in all four subregions) (Figure 2a), followed by the black‐naped tern (*Sterna sumatrana*, 43 sites) and the brown noddy (*Anous stolidus*, 38) (Figure 2b). Although 12 of the 15 species were present in both remote (Chesterfield archipelago and d'Entrecasteaux reefs) and mainland CRIs (northern and southwestern lagoons), most concentrated the majority of their breeding sites and populations in only one of the two groups of islands (Table 1; Figure 2b,c). Among the remaining three species, the silver gull was only found on mainland CRIs, and both the great and lesser frigatebirds (*Fregata ariel* and *Fregata minor*) bred exclusively on remote islands (Table 1; Figure 2d). The spatial segregation of species assemblages was further confirmed by the statistical comparison of breeding communities, which showed significant differences between mainland and remote CRIS (ANOSIM, *p* < 0.05) (Figure 3). Differences were also observed between subregions because the southwestern lagoon had a set of breeding species distinct from the other three subregions, which included all nine species of Laridae present in the study area (Figure 3; Appendix S8).

### Influence of habitat availability on species presence and abundance

**FIGURE 4:**
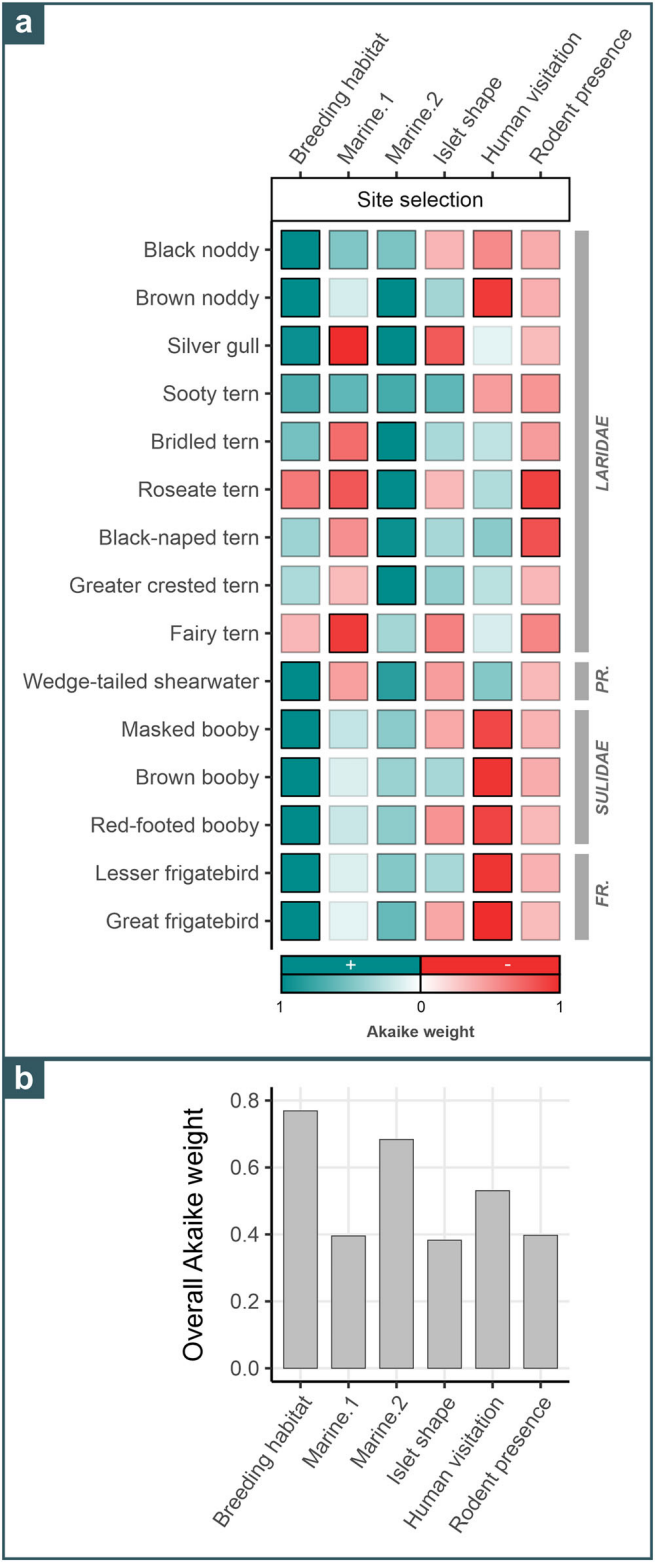
(a) Comparison of Akaike information criterion weights for six predictors used to model seabird site selection (presence is the binomial response variable in logit link, generalized linear model; value near 1, strong effect of corresponding predictor; value near 0, absence of effect; blue‐green, overall positive effect on the response variable; red, overall negative effect on the response variable; effects, based on weighted average of each predictor's coefficients across all tested models) and (b) Akaike weights of each predictor averaged across all species (overall weight) (*n* = 15; FR, Fregatidae; PR, Procellariidae; marine.1 and marine.2, first and second dimensions of a principal component analysis of neighboring geographic features in a 200‐km radius around study sites).

**FIGURE 5:**
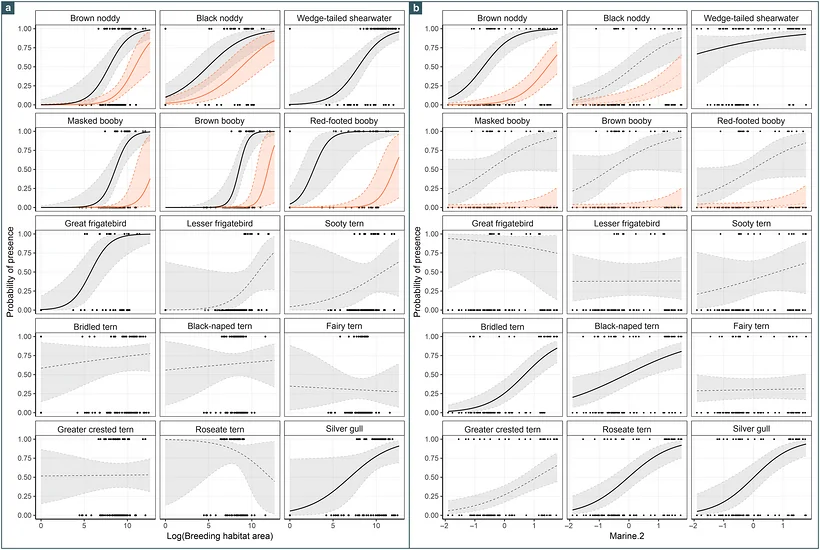
Observed (dots) and predicted probabilities (black lines) of seabird species presence with (a) area of breeding habitat and (b) marine.2 (second dimension of a principal component analysis of neighboring geographic features in a 200‐km radius around study sites) as predictors (gray shading, 95% prediction intervals; solid lines, significant effect of the corresponding predictor; when statistically significant, effect of human visitation is shown with predicted presence probabilities for the 0.75 quantile [high visitation] of the visitation predictor [orange lines and ribbons]).

Among all tested predictors, the area of breeding habitat had the highest overall influence on species occurrence and displayed a significant effect in eight of the 15 considered species (Figures 4a,b and 5a). Greater habitat availability was associated with increased probability of presence (*p* < 0.05) for the two species of noddies, the three species of boobies (masked booby [*Sula dactylatra*], brown booby [*S. leucogaster*], and red‐footed booby [*S. sula*]), the great frigatebird, the wedge‐tailed shearwater, and the silver gull (Figure 5a; Appendix S9). However, breeding habitat area did not appear to influence the presence of most Laridae species outside noddies. Akaike weights of the predictor were much lower (Figure 4a), and its effect on presence was evaluated as nonsignificant in the averaged model (Figure 5a; Appendix S9). Among species whose presence responded to habitat availability, black and brown noddies displayed lower inflexion thresholds (habitat area above which the probability of presence increases) than masked and brown boobies (Figure 5a).

**FIGURE 6:**
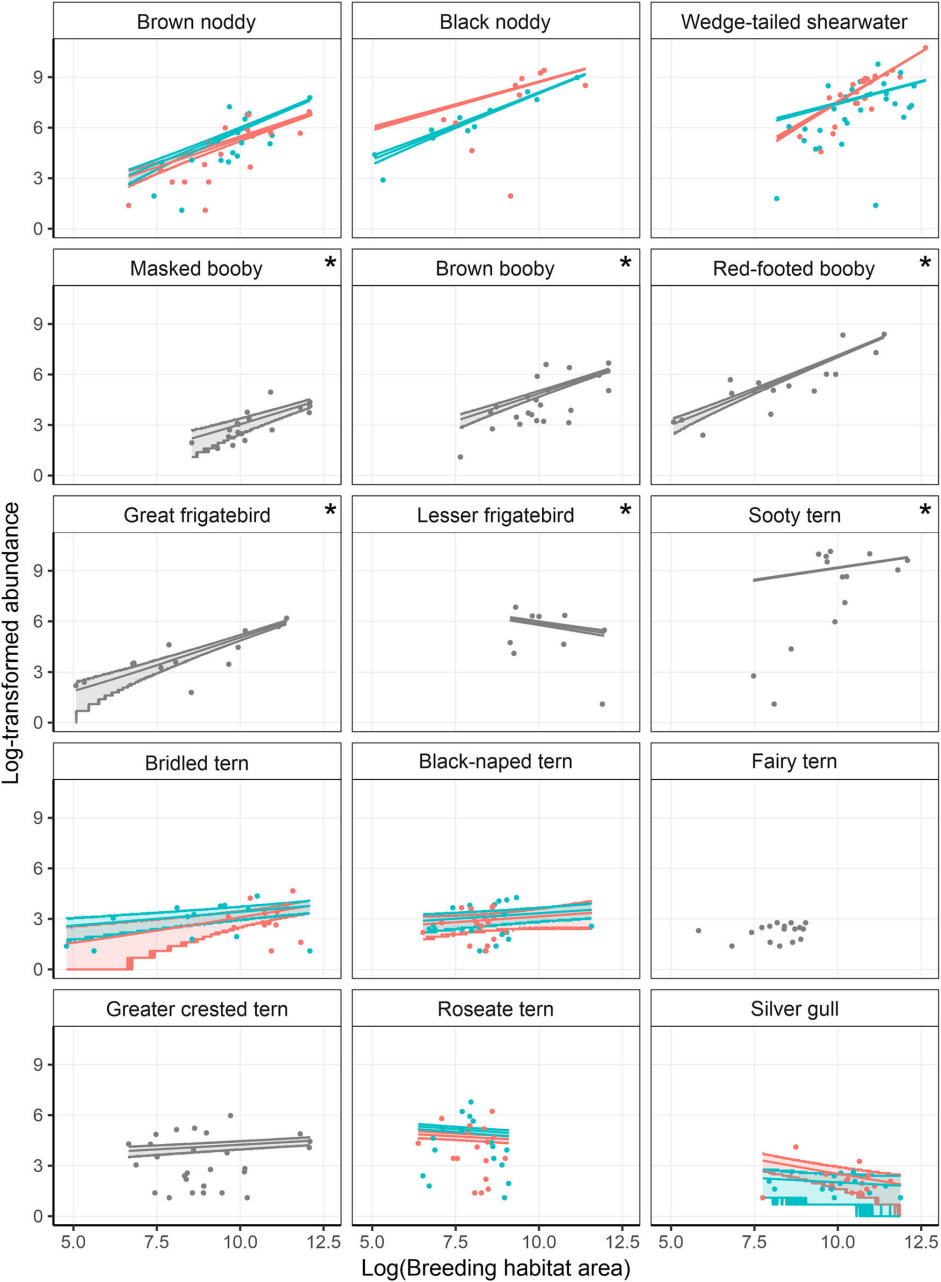
Observed (dots) and predicted (lines) values of seabird species abundance with area of breeding habitat as the predictor (shading, 95% prediction intervals; *, species not tested because almost all sites received comparably low visitation; when significant, the effect of human visitation is shown for [red] ≥median level of visitation of coral reef islands [CRIs] and for [blue] <median level of visitation of CRIs; predictions obtained from models described in Appendix S10).

On sites hosting breeding populations, abundances were significantly influenced by the area of breeding habitat for a higher proportion of species (14 of 15), displaying a positive effect for 11 taxa (all noddies and boobies, great frigatebird, sooty and bridled [*Onychoprion anaethetus*] terns, greater‐crested tern [*Thalasseus bergii*], black‐naped tern, and the wedge‐tailed shearwater) (Figure 6; Appendix S10). For lesser frigatebird, roseate tern, and silver gull, increase in breeding habitat area was linked to lower abundances, albeit with the smallest effect sizes among all species. Consequently, although site selection for the silver gull was favored by habitat availability (Figure 5), its abundance was not. For seven species (all noddies and boobies, great frigatebird, and wedge‐tailed shearwater), habitat availability positively influenced presence and abundance. Conversely, neither the presence nor the abundance of fairy terns appeared to be influenced by breeding habitat area (Appendices S9 & S10).

### Influence of neighboring geographical features

Geographical isolation from the mainland (approximated by marine.1 predictor, which effectively separates mainland and remote sites) outweighed the influence of human visitation on species presence in nine taxa (Appendix S11), for which only the former predictor was retained in the final averaged models (Appendix S9). A significant effect of isolation was observed in five species, among which most species of neritic Laridae (bridled, black‐naped, and fairy terns and silver gull) were more likely to be present on mainland CRIs (Figure 4a). Conversely, sooty terns were significantly more likely to breed on remote sites. The synthetic marine.2 predictor ranked second in overall influence on species presence (Figure 4b) and was the only predictor that had a positive effect across all species (Figure 4a). It significantly influenced the presence probability of most neritic species of terns, except the fairy and greater crested tern, and of the brown noddy and wedge‐tailed shearwater (Figure 5b; Appendix S9). For these taxa, high predictor values, corresponding to sites with a high proportion of lagoon up to 50 km from the shoreline, were associated with increased probability of presence. Apart from the greater‐crested and fairy terns, all species whose presence was not significantly influenced by the area of breeding habitat responded to at least one of the two geographical predictors.

### Effects of human disturbance and rodent presence

A high level of human disturbance, as approximated by the Visitation predictor, significantly decreased the probability of presence for all species of boobies and noddies (five species) (Figures 4a and 5a; Appendix S9) but did not appear to influence site selection for the remaining taxa. Visitation had a more varied influence on abundance: four species (black‐naped, bridled and roseate terns, and brown noddy) displayed decreased abundances in highly visited sites and two (black noddy and silver gull) showed the opposite trend (Figure 6; Appendix S10). Effect of visitation on wedge‐tailed shearwater abundances differed between small (negative influence of visitation) and large CRIs (seemingly positive influence) (Figure 6).

Rodent presence appeared to have little influence on site selection but did negatively affect wedge‐tailed shearwater abundance (*p* < 0.001) (Appendix S10). Because the latter was present on 13 of the rodent‐infested islets, we compared breeding densities (in number of breeding pairs per surface unit of breeding habitat) between sites with or without rodent populations. Breeding density was noticeably lower in rodent‐infested sites (mean [SD] = 4.5 breeding pairs/100 m^2^ [3.4], *n* = 13) than in rodent‐free sites (8.0 ± 6.8, *n* = 45) and minimal in CRIs that hosted black rats (2.7 ± 2.2, *n* = 4) (Appendix S12), although without significant differences (Wilcoxon test, *p* > 0.05).

## DISCUSSION

### Species responses as they relate to taxonomic and ecological traits

Differences in species assemblages across mainland and remote CRIs broadly reflected the concentration of short‐range neritic foragers (silver gull and all terns except the sooty tern and noddies) near the mainland and pelagic foragers (boobies and frigatebirds) in isolated CRIs (Catry, Ramos, Jaquemet, et al., 2009; Weimerskirch et al., 2005). Although the diet and foraging distances of tern species has not been previously documented in New Caledonia, assessments conducted on other tropical islands suggest that most tropical terns forage in shallow waters within 30 km of their breeding sites and conduct multiple trips per day during the breeding season (McLeay et al., 2010; Monticelli et al., 2008; Surman & Wooller, 2003). Around New Caledonia, the observed influence of the geographical predictors (marine.1 and marine.2) on tern presence suggests that optimal breeding sites for terns are those with the highest proportion of lagoon waters within 50 km of their shoreline, which likely offer the best resource availability for breeders (Appendix S5). Habitat availability did not affect tern presence, likely because their preferred habitats (bare ground or areas with low plant cover close to the shore) are present on most of the considered CRIs and therefore do not act as a discriminating factor (Batianoff, 2000; Berr, Millon, et al., 2023; Carr, Trevail, et al., 2021). This finding is consistent with those from previous surveys of tern populations in New Caledonia (excluding noddies) that show low fidelity and high interannual variation in the selection of breeding sites, except for the bridled tern (Pandolfi‐Benoit & Bretagnolle, 2002). Among neritic feeders, only silver gull presence was affected by both habitat presence and resource availability, but this species uses different nesting habitat (mostly under low‐lying shrubs) from most terns, which favor open, unvegetated areas (Appendix S4).

The distribution of most offshore and pelagic feeders (boobies, frigatebirds, sooty tern, and wedge‐tailed shearwater) appeared to be less constrained by geographic drivers than neritic species (Figures 4, 5, 6; Appendices S9 & S11). The former have long foraging distances, with typical trips within a 50–100 km range from breeding sites for masked boobies (Sommerfeld et al., 2015); >100 km for sooty terns, red‐footed boobies, and brown boobies (Jaquemet et al., 2008; Soanes et al., 2021; Weimerskirch et al., 2005); and up to 1000 km for frigatebirds and wedge‐tailed shearwaters (Weimerskirch et al., 2004, 2010, 2020). Foraging distances for these last species are above the range of the areas considered around CRIs (0–200 km radius), which likely explains the nonsignificance of geographical predictors in their respective models. Red‐footed boobies and sooty terns can exhibit different foraging strategies or diets depending on the location of studied populations, which also suggests they can adapt to diverse food resources and environmental constraints (Jaquemet et al., 2008; Mendez et al., 2017). Therefore, their presence on CRIs appeared less constrained by neighboring resource accessibility than that of short‐range foragers within the study area. However, resource availability influences their distribution at wider geographic scales, where ocean productivity and environmental drivers vary across larger ranges (Catry, Ramos, Le Corre, et al., 2009; Mendez et al., 2017).

The observed influence of specific habitat availability on seabird presence indicated that offshore and pelagic feeders tended to breed on most sites with sufficient breeding habitat, and the positive effect of habitat area on abundance showed that they used most of the available space on their breeding sites (Figure 4; Appendices S9 & S10). The considered species used a variety of habitats (Appendix S4): mixed shrubs for black noddies, red‐footed boobies, and great frigatebirds; bare ground or low vegetation for brown and masked boobies and lesser frigatebirds and brown noddies; and all habitats except beach areas for the burrow‐nesting wedge‐tailed shearwater. For those species, the strong relationship between breeding habitat area and species presence and abundance validates the habitat categories we selected, which have been used in prior assessments of habitat preferences in CRI seabirds (Batianoff & Cornelius, 2005; Carr, Trevail, et al., 2021). Brown noddies were the only offshore feeders for which site selection was influenced by both habitat and geographical drivers, which suggests a higher level of constraint on their spatial distribution.

### Avoidance of human disturbance

Berr, Millon, et al. (2023) observed a negative influence of human visitation on the species richness and biomass of seabird communities in our study area. Our focus on individual species responses showed that five of the 15 considered species were less likely to be present on highly visited CRIs, and four displayed decreased abundances (respectively reducing overall species richness and biomass). This result suggests that human disturbance caused by frequent visitation (trips by tourists, local campers, and fishers) reduces overall site attractivity even without severe impacts (e.g., egg harvesting or poaching), causing the species most sensitive to regular visitation to be absent from otherwise suitable breeding sites. When species are present, disturbance diminishes breeding densities within the available habitat, leading to fewer breeders compared with more secluded CRIs with a similar suitable area. The latter can imply that highly visited sites host colonies that use only the least‐disturbed areas of the available habitat (intrahabitat constraints on nest‐site selection) (Carney & Sydeman, 1999; Devney & Congdon, 2009) or that human disturbance selects against the individuals of a species most sensitive to disturbance or both (Thibault et al., 2020). The absence of a negative impact of visitation on the remaining set of species could partly reflect a low level of overall disturbance (only 10 CRIs host permanent tourist infrastructure). Such an observation may, however, be specific to New Caledonia, whose islands are less exposed to anthropogenic pressure than other tropical insular regions (e.g., Maldives and Cayman islands) (Cinner et al., 2018; Maire et al., 2016).

Site selection for all three species of boobies (masked, brown, and red‐footed boobies) and both species of noddies (black and brown noddies) was negatively affected by visitation, as was the abundance of black‐naped, roseate and bridled terns, and the brown noddy. Masked and brown boobies and the tern species nest on the ground and are generally concentrated in peripheral areas of CRIs (Batianoff & Cornelius, 2005; Borsa et al., 2010; Weimerskirch et al., 2009), which makes them especially vulnerable to disturbance by human visitors (nest trampling, induction of flight response in incubating birds and chicks, and colony desertion) (Burger & Gochfeld, 1993; Carney & Sydeman, 1999). Nesting behaviors of brown noddies on remote CRIs, where they mostly nest on the ground (Baudat‐Franceschi, 2011), differ from their nesting behaviors on mainland CRIs, where they primarily nest atop bay cedar bushes that are less exposed to visitor‐induced disturbances (Berr, Millon, et al., 2023; Pandolfi‐Benoit & Bretagnolle, 2002). This difference may reflect a behavioral adaptation to a higher level of disturbance on mainland CRIs but could also be the result of spatial competition with other ground nesters (e.g., roseate and greater crested terns) that are less abundant on remote CRIs. Negative impacts of disturbance on the presence of red‐footed boobies and black noddies were less straightforward because both species use octopus bush trees and *Pisonia* trees for nesting, which generally puts them out of the direct reach of visitors (Batianoff, 2000; Carret, Trevail, et al., 2021). We hypothesize that the local population size of both species is substantially lower than the carrying capacity offered by suitable breeding sites, allowing breeders to occupy CRIs with only the lowest level of disturbance. Both the red‐footed booby and the black noddy are able to nest on sites exposed to higher visitation in the absence of alternative, secluded breeding locations, as seen in CRIs from the Wallis‐et‐Futuna archipelago (Thibault et al., 2015). The apparent positive influence of visitation on the abundance of black noddies requires further investigation. It may be explained by the higher quality of available habitat on mainland breeding sites (dominated by *Pisonia* forests), which artificially link high densities of breeders with increased visitation, than that on remote CRIs (dominated by octopus bush trees). Finally, the area‐dependent effect of visitation observed for the wedge‐tailed shearwater (negative influence in small CRIs and seemingly positive for large sites) suggests that the effect of disturbance is strong on small CRIs whose entire surface is accessible to visitors but becomes negligible at large sites where only a small proportion of island surface is actually subject to visitation.

Although we observed no effect of human disturbance on the presence of most tern species, the human disturbance proxy we used gives only a broad measure of site exposure to disturbance and does not reflect direct impacts on breeding success or survival of seabirds. The absence of influence in terns indicated that they nest indifferently on sites with high and low visitation; however, the literature contains no evidence suggesting that they withstand human disturbance better than other species. On the contrary, prior examples show that human disturbance reduces breeding success in roseate and black‐naped terns, either through direct impacts (Pandolfi‐Benoit & Bretagnolle, 2002) or through nest predation by gulls or rails after adults have fled from intruders (King et al., 1992). Because resource accessibility appeared to strongly constrain site selection in terns, it is likely that they are less able to relocate to sites with low disturbance than offshore and pelagic feeders. In New Caledonia, mitigation of human impacts on terns is ongoing in the southwestern lagoon since 2014 and in the northern lagoon since 2018. The mitigation involves temporary access restrictions to sites that host tern colonies during the austral summer (black‐naped, roseate, and greater crested terns) and winter (fairy tern) breeding seasons. However, the effects of these restrictions on breeding success have so far not been monitored.

Significant impacts of rodent presence on colony size were observed for the burrow‐nesting wedge‐tailed shearwater. Such a result is consistent with documented negative effects of rodents on seabird demography, suggesting that burrow and ground nesters are the species most vulnerable to predation by invasive rodents (Marie et al., 2014; VanderWerf et al., 2014). Although inconclusive due to small sample size, our qualitative comparison of wedge‐tailed shearwater densities between black rat‐infested and rodent‐free sites (Appendix S12) is also consistent with known impacts of black rats, whose effects on seabirds are the most severe compared with those of other rodent species (Shiels et al., 2014). Rodents had no effect on other seabirds, but our dataset offered limited power for assessing negative impacts of rodents due to the rarity of rodent‐infested sites among the considered CRIs (15 sites); in addition, our lumping of several species into a single binary predictor of rodent presence could have masked species‐specific effects. Because long‐term monitoring data were lacking, we could not address possible long‐term demographic consequences of eradications on sites that previously hosted rodent populations (Baudat‐Franceschi et al., 2008; Bell, 1998).

### Lessons for future management

In a context of possible CRI decline and rarefaction of available breeding sites due to expected SLR and coastal erosion, our results offer valuable insight into future conservation planning for CRI seabirds. In particular, management strategies should separate functional groups of seabirds that have different vulnerabilities and restoration potential.

Site selection in offshore and pelagic‐feeding seabirds appears to be mostly driven by habitat availability and isolation from human disturbance. Consequently, one can expect these species to have high relocation and recolonization potential, as long as their resources in pelagic environments are not depleted (Cury et al., 2011) and their breeding habitats are maintained. They may therefore be at lower risk of demographic decline than short‐range foragers in the event of a partial disappearance of breeding sites (Reynolds et al., 2015). Habitat management through the restoration of native plant species (savanna with low plant cover and mixed shrubs of *Pisonia* and octopus bush tree) (Carr, Trevail, et al., 2021; Feare et al., 2015), the eradication of introduced mammals (Philippe‐Lesaffre et al., 2023), and the mitigation of human disturbance through access restrictions (Devney & Congdon, 2009) hold great potential for the restoration and possible relocation of seabird colonies, especially in association with social attraction efforts (Herrera‐Giraldo et al., 2021). However, high levels of philopatry may still hinder or slow translocation initiatives across CRIs (Danckwerts et al., 2021; Oro et al., 2011; Spatz et al., 2023).

Short‐range foragers that rely on CRIs for breeding and on lagoon resources for feeding are likely the species most vulnerable to the future disruption of reef ecosystems because they could be threatened simultaneously in their marine and terrestrial environments (Berr, Dias, et al., 2023; Perry et al., 2011). Their potential for relocation may be restricted to small‐scale areas in the vicinity of their current breeding sites, which offers fewer options to managers. Fine‐scale characterization of species biogeography and resource availability and the evaluation of projected impacts of coastal erosion are therefore necessary steps for orienting future conservation strategies.

### Prospects

Future development of the type of work we undertook includes the evaluation of more precise environmental drivers of resource (ocean productivity, depth, slope, and typology of reef habitats) and habitat availability (additional habitat categories, distribution of plant cover, etc.). Short‐range foragers respond to fine‐scale ecological cues in the marine environment (Pratte et al., 2021), and it was recently shown that nest distribution of tropical Laridae species, whose site selection was not influenced by our broad habitat typology, was shaped by microscale ecological parameters (Garcia‐Quintas, Denis, et al., 2023). Consequently, we believe that a more accurate understanding of site selection and onsite distribution in short‐range foragers is a priority for future study of CRI seabirds because they are likely more sensitive to coastal erosion and submersion hazards. Developing similar studies at broader geographic scales would improve understanding of large‐scale distribution patterns of CRI seabirds, which is necessary for international management efforts (Berr, Millon, et al., 2023; https://www.sprep.org/ioe/regional‐marine‐species‐programme). New Caledonia seabird species almost all occur in other tropical archipelagos with different geographic, environmental, and anthropogenic contexts (e.g., Chagos archipelago, Great Barrier Reef, and French Polynesia), which reinforces the importance of interregional comparisons of seabird biogeography (Carr, Votier, et al., 2021; Congdon et al., 2007; Thibault & Bretagnolle, 2007).

## Supporting information



Supporting information

## References

[cobi70335-bib-0001] Álvarez‐Romero, J. G. , Pressey, R. L. , Ban, N. C. , Vance‐Borland, K. , Willer, C. , Klein, C. J. , & Gaines, S. D. (2011). Integrated land‐sea conservation planning: The missing links. Annual Review of Ecology, Evolution and Systematics, 42, 381–409. 10.1146/annurev-ecolsys-102209-144702

[cobi70335-bib-0002] Aslam, M. , & Kench, P. S. (2017). Reef island dynamics and mechanisms of change in Huvadhoo Atoll, Republic of Maldives, Indian Ocean. Anthropocene, 18, 57–68. 10.1016/j.ancene.2017.05.003

[cobi70335-bib-0003] Bartoń, K. (2023). MuMIn: Multi‐model inference (Version 1.47.5) [Computer software]. https://cran.r‐project.org/web/packages/MuMIn/index.html

[cobi70335-bib-0004] Batianoff, G. N. (2000). Utilisation of seashore vegetation by tropical seabirds on North East Cay (Herald Lays), Coringa‐Herald National Nature Reserve, Australia. The Sunbird: Journal of the Queensland Ornithological Society, 30(1), 1–17. https://search.informit.org/doi/10.3316/informit.911874275363275

[cobi70335-bib-0005] Batianoff, G. N. , & Cornelius, N. J. (2005). Birds of Raine island: Population trends, breeding behaviour and nesting habitats. The Proceedings of the Royal Society of Queensland, 112, 1–29. https://search.informit.org/doi/10.3316/ielapa.176416379085803

[cobi70335-bib-0006] Baudat‐Franceschi, J. (2011). Oiseaux: Diversité spécifique, populations, enjeux de conservation. In E. Clua , L. Gardes , S. A. McKenna , & C. Vieux (Eds.), Contribution à l'inventaire biologique et à l’évaluation des ressources sur les récifs des Chesterfield (pp. 157–180). SPREP.

[cobi70335-bib-0007] Baudat‐Franceschi, J. , Spaggiari, J. , & Barré, N. (2008). Oiseaux Nicheurs d'Intérêt Pour la Conservation. In S. A. McKenna , N. Baillon , & J. Spaggiari (Eds.), A rapid marine biodiversity assessment of the coral reefs of the Northwest Lagoon, between Koumac and Yandé, Province Nord, New Caledonia (pp. 136–142). Conservation International. 10.1896/054.053.0114

[cobi70335-bib-0008] Bell, M. (1998). Seabird island restoration: New Caledonia; Eradication of rats, October 1998. Wildlife Management International—Direction des Ressources Naturelles de Nouvelle‐Calédonie.

[cobi70335-bib-0009] Benjamini, Y. , & Hochberg, Y. (1995). Controlling the false discovery rate: A practical and powerful approach to multiple testing. Journal of the Royal Statistical Society Series B: Statistical Methodology, 57(1), 289–300. 10.1111/j.2517-6161.1995.tb02031.x

[cobi70335-bib-0010] Benkwitt, C. E. , D'Angelo, C. , Dunn, R. E. , Gunn, R. L. , Healing, S. , Mardones, M. L. , Wiedenmann, J. , Wilson, S. K. , & Graham, N. A. J. (2023). Seabirds boost coral reef resilience. Science Advances, 9(49), Article eadj0390. 10.1126/sciadv.adj0390 PMC10699780 38055814

[cobi70335-bib-0011] Benkwitt, C. E. , Gunn, R. L. , Le Corre, M. , Carr, P. , & Graham, N. A. J. (2021). Rat eradication restores nutrient subsidies from seabirds across terrestrial and marine ecosystems. Current Biology, 31(12), 2704.e4–2711.e4. 10.1016/j.cub.2021.03.104 33887185

[cobi70335-bib-0012] Benoit, M. P. , & Bretagnolle, V. (2002). Seabirds of the Southern Lagoon of New Caledonia; Distribution, abundance and threats. Waterbirds: The International Journal of Waterbird Biology, 25(2), 202–213.

[cobi70335-bib-0013] Berr, T. , Dias, M. P. , Andréfouët, S. , Davies, T. , Handley, J. , Le Corre, M. , Millon, A. , & Vidal, É. (2023). Seabird and reef conservation must include coral islands. Trends in Ecology & Evolution, 38(6), 490–494. 10.1016/j.tree.2023.02.004 36925406

[cobi70335-bib-0014] Berr, T. , Millon, A. , Dumas, P. , Guehenneuc, P. , Perez, F. , De Méringo, H. , Baudat‐Franceschi, J. , Le Corre, M. , & Vidal, É. (2023). Human visitation disrupts natural determinants of breeding seabird communities on coral reef islands. Global Ecology and Conservation, 48, Article e02732. 10.1016/j.gecco.2023.e02732

[cobi70335-bib-0015] Berr, T. , Naudet, J. , Lagourgue, C. , Vuibert, K. , Bourgeois, K. , & Vidal, É. (2020). Plastic ingestion by seabirds in New Caledonia, South Pacific. Marine Pollution Bulletin, 152, Article 110925. 10.1016/j.marpolbul.2020.110925 32479297

[cobi70335-bib-0101] BirdLife International . (2026). IUCN Red List for birds. https://datazone.birdlife.org

[cobi70335-bib-0016] Bivand, R. , Altman, M. , Anselin, L. , Assunção, R. , Berke, O. , Blanchet, F. G. , Carvalho, M. , Christensen, B. , Chun, Y. , Dormann, C. , Dray, S. , Dunnington, D. , Gómez‐Rubio, V. , Krainski, E. , Legendre, P. , Lewin‐Koh, N. , Li, A. , Millo, G. , Mueller, W. , … Yu, D. (2023). spdep: Spatial dependence: Weighting schemes, statistics (Version 1.2‐8) [Computer software]. https://cran.r‐project.org/web/packages/spdep/index.html

[cobi70335-bib-0017] Borsa, P. , Pandolfi, M. , Andréfouët, S. , & Bretagnolle, V. (2010). Breeding avifauna of the Chesterfield Islands, Coral Sea: Current population sizes, trends, and threats. Pacific Science, 64(2), 297–314. 10.2984/64.2.297

[cobi70335-bib-0018] Borsa, P. , & Vidal, E. (2018). Vulnerable and threatened: The seabirds of the Coral Sea. In C. E. Payri (Ed.), New Caledonia—World of corals (pp. 135–140). IRD Éditions/Solaris.

[cobi70335-bib-0019] Burger, J. , & Gochfeld, M. (1993). Tourism and short‐term behavioural responses of nesting masked, red‐footed, and blue‐footed, boobies in the Galápagos. Environmental Conservation, 20(3), 255–259. 10.1017/S0376892900023043

[cobi70335-bib-0020] Burnham, K. P. , & Anderson, D. R. (Eds.). (2004). Model selection and multimodel inference. Springer. 10.1007/b97636

[cobi70335-bib-0021] Carlson, R. R. , Evans, L. J. , Foo, S. A. , Grady, B. W. , Li, J. , Seeley, M. , Xu, Y. , & Asner, G. P. (2021). Synergistic benefits of conserving land‐sea ecosystems. Global Ecology and Conservation, 28, Article e01684. 10.1016/j.gecco.2021.e01684

[cobi70335-bib-0022] Carney, K. M. , & Sydeman, W. J. (1999). A review of human disturbance effects on nesting colonial waterbirds. Waterbirds: The International Journal of Waterbird Biology, 22(1), 68–79. 10.2307/1521995

[cobi70335-bib-0023] Carr, P. , Trevail, A. , Bárrios, S. , Clubbe, C. , Freeman, R. , Koldewey, H. J. , Votier, S. C. , Wilkinson, T. , & Nicoll, M. A. C. (2021). Potential benefits to breeding seabirds of converting abandoned coconut plantations to native habitats after invasive predator eradication. Restoration Ecology, 29(5), Article 5. 10.1111/rec.13386

[cobi70335-bib-0024] Carr, P. , Votier, S. , Koldewey, H. , Godley, B. , Wood, H. , & Nicoll, M. a. C. (2021). Status and phenology of breeding seabirds and a review of Important Bird and Biodiversity Areas in the British Indian Ocean Territory. Bird Conservation International, 31(1), 14–34. 10.1017/S0959270920000295

[cobi70335-bib-0025] Catry, T. , Ramos, J. , Jaquemet, S. , Faulquier, L. , Berlincourt, M. , Hauselmann, A. , Pinet, P. , & Le Corre, M. (2009). Comparative foraging ecology of a tropical seabird community of the Seychelles, western Indian Ocean. Marine Ecology Progress Series, 374, 259–272. 10.3354/meps07713

[cobi70335-bib-0026] Catry, T. , Ramos, J. , Le Corre, M. , & Phillips, R. (2009). Movements, at‐sea distribution and behaviour of a tropical pelagic seabird: The wedge‐tailed shearwater in the western Indian Ocean. Marine Ecology Progress Series, 391, 231–242. 10.3354/meps07717

[cobi70335-bib-0027] Caut, S. , Angulo, E. , & Courchamp, F. (2009). Avoiding surprise effects on Surprise Island: Alien species control in a multitrophic level perspective. Biological Invasions, 11(7), 1689–1703. 10.1007/s10530-008-9397-9

[cobi70335-bib-0028] Cinner, J. E. , Maire, E. , Huchery, C. , MacNeil, M. A. , Graham, N. A. J. , Mora, C. , McClanahan, T. R. , Barnes, M. L. , Kittinger, J. N. , Hicks, C. C. , D'Agata, S. , Hoey, A. S. , Gurney, G. G. , Feary, D. A. , Williams, I. D. , Kulbicki, M. , Vigliola, L. , Wantiez, L. , Edgar, G. J. , … Mouillot, D. (2018). Gravity of human impacts mediates coral reef conservation gains. Proceedings of the National Academy of Sciences, 115(27), E6116–E6125. 10.1073/pnas.1708001115 PMC6142230 29915066

[cobi70335-bib-0029] Clay, P. M. , Howard, J. , Busch, D. S. , Colburn, L. L. , Himes‐Cornell, A. , Rumrill, S. S. , Zador, S. G. , & Griffis, R. B. (2020). Ocean and coastal indicators: Understanding and coping with climate change at the land‐sea interface. Climatic Change, 163(4), 1773–1793. 10.1007/s10584-020-02940-x

[cobi70335-bib-0030] Congdon, B. C. , Erwin, C. A. , Peck, D. R. , Baker, G. B. , Double, M. C. , & O'Neill, P. (2007). Vulnerability of seabirds on the Great Barrier Reef to climate change. In J. Johnson & P. Marshall (Eds.), Climate change and the Great Barrier Reef: A vulnerability assessment (pp. 428–463). The Great Barrier Reef Marine Park Authority. https://elibrary.gbrmpa.gov.au/jspui/handle/11017/546

[cobi70335-bib-0031] Cornwall, C. E. , Comeau, S. , Kornder, N. A. , Perry, C. T. , van Hooidonk, R. , DeCarlo, T. M. , Pratchett, M. S. , Anderson, K. D. , Browne, N. , Carpenter, R. , Diaz‐Pulido, G. , D'Olivo, J. P. , Doo, S. S. , Figueiredo, J. , Fortunato, S. A. V. , Kennedy, E. , Lantz, C. A. , McCulloch, M. T. , González‐Rivero, M. , … Lowe, R. J. (2021). Global declines in coral reef calcium carbonate production under ocean acidification and warming. Proceedings of the National Academy of Sciences, 118(21), Article e2015265118. 10.1073/pnas.2015265118 PMC8166140 33972407

[cobi70335-bib-0032] Cury, P. M. , Boyd, I. L. , Bonhommeau, S. , Anker‐Nilssen, T. , Crawford, R. J. M. , Furness, R. W. , Mills, J. A. , Murphy, E. J. , Österblom, H. , Paleczny, M. , Piatt, J. F. , Roux, J.‐P. , Shannon, L. , & Sydeman, W. J. (2011). Global seabird response to forage fish depletion—One‐Third for the birds. Science, 334(6063), 1703–1706. 10.1126/science.1212928 22194577

[cobi70335-bib-0033] D'agata, S. , Mouillot, D. , Kulbicki, M. , Andréfouët, S. , Bellwood, D. R. , Cinner, J. E. , Cowman, P. F. , Kronen, M. , Pinca, S. , & Vigliola, L. (2014). Human‐mediated loss of phylogenetic and functional diversity in coral reef fishes. Current Biology, 24(5), 555–560. 10.1016/j.cub.2014.01.049 24560574

[cobi70335-bib-0034] Danckwerts, D. K. , Humeau, L. , Pinet, P. , McQuaid, C. D. , & Le Corre, M. (2021). Extreme philopatry and genetic diversification at unprecedented scales in a seabird. Scientific Reports, 11(1), Article 6834. 10.1038/s41598-021-86406-9 PMC7994906 33767313

[cobi70335-bib-0035] Devney, C. A. , & Congdon, B. C. (2009). Testing the efficacy of a boundary fence at an important tropical seabird breeding colony and key tourist destination. Wildlife Research, 36(4), 353–360. 10.1071/WR08143

[cobi70335-bib-0036] Espíndola, W. D. , & Carlo, T. A. (2024). Seabird guano inputs increase impacts from introduced mammals on the native plants and animals of an oceanic island. Oecologia, 204(4), 975–984. 10.1007/s00442-024-05546-7 38597960

[cobi70335-bib-0037] Feare, C. J. , French, G. C. A. , Nevill, J. E. G. , Pattison‐Willits, V. S. , Wheeler, V. , Yates, T. L. , Hoareau, C. , & Prescott, C. (2015). Attempted re‐establishment of a sooty tern *Onychoprion fuscatus* breeding colony on Denis Island, Seychelles. Conservation Evidence, 12, 19–24.

[cobi70335-bib-0038] Fernández‐Palacios, J. M. , Kreft, H. , Irl, S. D. H. , Norder, S. , Ah‐Peng, C. , Borges, P. A. V. , Burns, K. C. , de Nascimento, L. , Meyer, J.‐Y. , Montes, E. , & Drake, D. R. (2021). Scientists’ warning—The outstanding biodiversity of islands is in peril. Global Ecology and Conservation, 31, Article e01847. 10.1016/j.gecco.2021.e01847 PMC8556160 34761079

[cobi70335-bib-0039] Garcia‐Quintas, A. , Denis, D. , Barbraud, C. , & Bertrand, S. L. (2023). Breeding microhabitat patterns among sympatric tropical larids. Marine Ornithology, 51(1), 97–107.

[cobi70335-bib-0040] Garcia‐Quintas, A. , Roy, A. , Barbraud, C. , Demarcq, H. , Denis, D. , & Lanco Bertrand, S. (2023). Machine and deep learning approaches to understand and predict habitat suitability for seabird breeding. Ecology and Evolution, 13(9), Article e10549. 10.1002/ece3.10549 PMC10505760 37727776

[cobi70335-bib-0041] Garcin, M. , Vendé‐Leclerc, M. , Maurizot, P. , Le Cozannet, G. , Robineau, B. , & Nicolae‐Lerma, A. (2016). Lagoon islets as indicators of recent environmental changes in the South Pacific—The New Caledonian example. Continental Shelf Research, 122, 120–140. 10.1016/j.csr.2016.03.025

[cobi70335-bib-0042] Gargominy, O. , Bouchet, P. , Pascal, M. , Jaffré, T. , & Tourneur, J.‐C. (1996). Conséquences des introductions d'espèces animales et végétales sur la biodiversité en Nouvelle‐Calédonie. Revue d'Écologie (La Terre et La Vie), 51(4), 375–402.

[cobi70335-bib-0043] Garrigue, C. , Derville, S. , & Bonneville, C. (2018). Searching for humpback whales two centuries post‐whaling: What is left in the Chesterfield‐Bellona archipelago? (Report number: SC67B/SH17). International Whaling Commission.

[cobi70335-bib-0044] Gilby, B. L. , Olds, A. D. , Connolly, R. M. , Stevens, T. , Henderson, C. J. , Maxwell, P. S. , Tibbetts, I. R. , Schoeman, D. S. , Rissik, D. , & Schlacher, T. A. (2016). Optimising land‐sea management for inshore coral reefs. PLoS ONE, 11(10), Article e0164934. 10.1371/journal.pone.0164934 PMC5072624 27764164

[cobi70335-bib-0045] Gonson, C. , Pelletier, D. , Gamp, E. , Preuss, B. , Jollit, I. , & Ferraris, J. (2016). Decadal increase in the number of recreational users is concentrated in no‐take marine reserves. Marine Pollution Bulletin, 107(1), 144–154. 10.1016/j.marpolbul.2016.04.007 27103423

[cobi70335-bib-0046] Graham, N. A. J. , Wilson, S. K. , Carr, P. , Hoey, A. S. , Jennings, S. , & MacNeil, M. A. (2018). Seabirds enhance coral reef productivity and functioning in the absence of invasive rats. Nature, 559(7713), 250–253. 10.1038/s41586-018-0202-3 29995864

[cobi70335-bib-0047] Grant, M. L. , Bond, A. L. , & Lavers, J. L. (2022). The influence of seabirds on their breeding, roosting and nesting grounds: A systematic review and meta‐analysis. Journal of Animal Ecology, 91(6), 1266–1289. 10.1111/1365-2656.13699 PMC9324971 35395097

[cobi70335-bib-0048] Gunn, R. L. , Benkwitt, C. E. , Graham, N. A. J. , Hartley, I. R. , Algar, A. C. , & Keith, S. A. (2023). Terrestrial invasive species alter marine vertebrate behaviour. Nature Ecology & Evolution, 7(1), 82–91. 10.1038/s41559-022-01931-8 PMC9834043 36604551

[cobi70335-bib-0049] Harris, L. R. , Bessinger, M. , Dayaram, A. , Holness, S. , Kirkman, S. , Livingstone, T.‐C. , Lombard, A. T. , Lück‐Vogel, M. , Pfaff, M. , Sink, K. J. , Skowno, A. L. , & Van Niekerk, L. (2019). Advancing land‐sea integration for ecologically meaningful coastal conservation and management. Biological Conservation, 237, 81–89. 10.1016/j.biocon.2019.06.020

[cobi70335-bib-0050] Hatfield, J. S. , Reynolds, M. H. , Seavy, N. E. , & Krause, C. M. (2012). Population dynamics of Hawaiian seabird colonies vulnerable to sea‐level rise. Conservation Biology, 26(4), 667–678. 10.1111/j.1523-1739.2012.01853.x 22624702

[cobi70335-bib-0051] Herrera‐Giraldo, J.‐L. , Figuerola‐Hernández, C. E. , Wolf, C. A. , Colón‐Merced, R. , Ventosa‐Febles, E. , Silander, S. , & Holmes, N. D. (2021). The use of social attraction techniques to restore seabird colonies on Desecheo Island, Puerto Rico. Ecological Solutions and Evidence, 2(2), Article e12058. 10.1002/2688-8319.12058

[cobi70335-bib-0052] Honig, S. E. , & Mahoney, B. (2016). Evidence of seabird guano enrichment on a coral reef in Oahu, Hawaii. Marine Biology, 163(2), Article 22. 10.1007/s00227-015-2808-4

[cobi70335-bib-0053] Hughes, D. J. , Alderdice, R. , Cooney, C. , Kühl, M. , Pernice, M. , Voolstra, C. R. , & Suggett, D. J. (2020). Coral reef survival under accelerating ocean deoxygenation. Nature Climate Change, 10(4), 296–307. 10.1038/s41558-020-0737-9

[cobi70335-bib-0054] Hughes, T. P. , Kerry, J. T. , Álvarez‐Noriega, M. , Álvarez‐Romero, J. G. , Anderson, K. D. , Baird, A. H. , Babcock, R. C. , Beger, M. , Bellwood, D. R. , Berkelmans, R. , Bridge, T. C. , Butler, I. R. , Byrne, M. , Cantin, N. E. , Comeau, S. , Connolly, S. R. , Cumming, G. S. , Dalton, S. J. , Diaz‐Pulido, G. , … Wilson, S. K. (2017). Global warming and recurrent mass bleaching of corals. Nature, 543(7645), 373–377. 10.1038/nature21707 28300113

[cobi70335-bib-0055] Husson, F. , Josse, J. , Le, S. , & Mazet, J. (2023). FactoMineR: Multivariate exploratory data analysis and data mining (Version 2.8) [Computer software]. https://cran.r‐project.org/web/packages/FactoMineR/index.html

[cobi70335-bib-0056] Jaquemet, S. , Potier, M. , Cherel, Y. , Kojadinovic, J. , Bustamante, P. , Richard, P. , Catry, T. , Ramos, J. A. , & Le Corre, M. (2008). Comparative foraging ecology and ecological niche of a superabundant tropical seabird: The sooty tern Sterna fuscata in the southwest Indian Ocean. Marine Biology, 155(5), 505–520. 10.1007/s00227-008-1049-1

[cobi70335-bib-0057] Jones, H. P. , Appoo, J. , Benkwitt, C. E. , Borrelle, S. B. , Dunn, R. E. , Epstein, H. E. , Fowlke, L. A. , Holmes, N. D. , Jeannot, L.‐L. , Malhi, Y. , Rankin, L. L. , Steibl, S. , Wails, C. N. , Will, D. J. , & Graham, N. A. (2025). The circular seabird economy is critical for oceans, islands and people. Nature Reviews Biodiversity, 1, 689–702. 10.1038/s44358-025-00099-w

[cobi70335-bib-0058] Kane, H. H. , & Fletcher, C. H. (2020). Rethinking reef island stability in relation to anthropogenic sea level rise. Earth's Future, 8(10), Article e2020EF001525. 10.1029/2020EF001525

[cobi70335-bib-0059] King, B. R. , Hicks, J. T. , & Cornelius, J. (1992). Population changes, breeding cycles and breeding success over six years in a seabird colony at Michaelmas Cay, Queensland. Emu ‐ Austral Ornithology, 92(1), 1–10. 10.1071/MU9920001

[cobi70335-bib-0060] Kleypas, J. , Allemand, D. , Anthony, K. , Baker, A. C. , Beck, M. W. , Hale, L. Z. , Hilmi, N. , Hoegh‐Guldberg, O. , Hughes, T. , Kaufman, L. , Kayanne, H. , Magnan, A. K. , Mcleod, E. , Mumby, P. , Palumbi, S. , Richmond, R. H. , Rinkevich, B. , Steneck, R. S. , Voolstra, C. R. , … Gattuso, J.‐P. (2021). Designing a blueprint for coral reef survival. Biological Conservation, 257, Article 109107. 10.1016/j.biocon.2021.109107

[cobi70335-bib-0061] Le Corre, M. , Danckwerts, D. K. , Ringler, D. , Bastien, M. , Orlowski, S. , Morey Rubio, C. , Pinaud, D. , & Micol, T. (2015). Seabird recovery and vegetation dynamics after Norway rat eradication at Tromelin Island, western Indian Ocean. Biological Conservation, 185, 85–94. 10.1016/j.biocon.2014.12.015

[cobi70335-bib-0062] Lorrain, A. , Houlbrèque, F. , Benzoni, F. , Barjon, L. , Tremblay‐Boyer, L. , Menkes, C. , Gillikin, D. P. , Payri, C. , Jourdan, H. , Boussarie, G. , Verheyden, A. , & Vidal, E. (2017). Seabirds supply nitrogen to reef‐building corals on remote Pacific islets. Scientific Reports, 7(1), Article 3721. 10.1038/s41598-017-03781-y PMC5473863 28623288

[cobi70335-bib-0063] Lukacs, P. M. , Burnham, K. P. , & Anderson, D. R. (2010). Model selection bias and Freedman's paradox. Annals of the Institute of Statistical Mathematics, 62(1), 117–125. 10.1007/s10463-009-0234-4

[cobi70335-bib-0064] Maire, E. , Cinner, J. , Velez, L. , Huchery, C. , Mora, C. , Dagata, S. , Vigliola, L. , Wantiez, L. , Kulbicki, M. , & Mouillot, D. (2016). How accessible are coral reefs to people? A global assessment based on travel time. Ecology Letters, 19(4), 351–360. 10.1111/ele.12577 26879898

[cobi70335-bib-0065] Marie, A. , VanderWerf, E. A. , Young, L. C. , Smith, D. G. , Eijzenga, J. , & Lohr, M. T. (2014). Response of wedge‐tailed shearwaters (*Puffinus pacificus*) to eradication of black rats (*Rattus rattus*) from Moku'auia Island after reinvasion. Pacific Science, 68(4), 547–553. 10.2984/68.4.8

[cobi70335-bib-0066] Masselink, G. , Beetham, E. , & Kench, P. (2020). Coral reef islands can accrete vertically in response to sea level rise. Science Advances, 6(24), Article eaay3656. 10.1126/sciadv.aay3656 PMC7286686 32577502

[cobi70335-bib-0067] McLeay, L. , Page, B. , Goldsworthy, S. , Paton, D. , Teixeira, C. , Burch, P. , & Ward, T. (2010). Foraging behaviour and habitat use of a short‐ranging seabird, the crested tern. Marine Ecology Progress Series, 411, 271–283. 10.3354/meps08606

[cobi70335-bib-0068] Mendez, L. , Borsa, P. , Cruz, S. , de Grissac, S. , Hennicke, J. , Lallemand, J. , Prudor, A. , & Weimerskirch, H. (2017). Geographical variation in the foraging behaviour of the pantropical red‐footed booby. Marine Ecology Progress Series, 568, 217–230. 10.3354/meps12052

[cobi70335-bib-0069] Monticelli, D. , Ramos, J. A. , Tavares, P. C. , Bataille, B. , Lepoint, G. , & Devillers, P. (2008). Diet and foraging ecology of roseate terns and lesser noddies breeding sympatrically on Aride Island, Seychelles. Waterbirds, 31(2), 231–240.

[cobi70335-bib-0070] Oro, D. , Martínez‐Abraín, A. , Villuendas, E. , Sarzo, B. , Mínguez, E. , Carda, J. , & Genovart, M. (2011). Lessons from a failed translocation program with a seabird species: Determinants of success and conservation value. Biological Conservation, 144(2), 851–858. 10.1016/j.biocon.2010.11.018

[cobi70335-bib-0071] Pandolfi, J. M. , Connolly, S. R. , Marshall, D. J. , & Cohen, A. L. (2011). Projecting coral reef futures under global warming and ocean acidification. Science, 333(6041), 418–422. 10.1126/science.1204794 21778392

[cobi70335-bib-0072] Pang, T. , Wang, X. , Nawaz, R. A. , Keefe, G. , & Adekanmbi, T. (2023). Coastal erosion and climate change: A review on coastal‐change process and modeling. Ambio, 52, 2034–2052. 10.1007/s13280-023-01901-9 PMC10654294 37405570

[cobi70335-bib-0073] Paytan, A. , Shellenbarger, G. G. , Street, J. H. , Gonneea, M. E. , Davis, K. , Young, M. B. , & Moore, W. S. (2006). Submarine groundwater discharge: An important source of new inorganic nitrogen to coral reef ecosystems. Limnology and Oceanography, 51(1), 343–348. 10.4319/lo.2006.51.1.0343

[cobi70335-bib-0074] Perry, C. T. , Kench, P. S. , Smithers, S. G. , Riegl, B. , Yamano, H. , & O'Leary, M. J. (2011). Implications of reef ecosystem change for the stability and maintenance of coral reef islands. Global Change Biology, 17(12), 3679–3696. 10.1111/j.1365-2486.2011.02523.x

[cobi70335-bib-0075] Philippe‐Lesaffre, M. , Thibault, M. , Caut, S. , Bourgeois, K. , Berr, T. , Ravache, A. , Vidal, E. , Courchamp, F. , & Bonnaud, E. (2023). Recovery of insular seabird populations years after rodent eradication. Conservation Biology, 37(3), Article e14042. 10.1111/cobi.14042 36661083

[cobi70335-bib-0076] Pratte, I. , Ronconi, R. , Craik, S. , & McKnight, J. (2021). Spatial ecology of endangered roseate terns and foraging habitat suitability around a colony in the western North Atlantic. Endangered Species Research, 44, 339–350. 10.3354/esr01108

[cobi70335-bib-0102] R Core Team . (2023). R: A language and environment for statistical computing. R Foundation for Statistical Computing, Vienna, Austria. https://www.R‐project.org/

[cobi70335-bib-0077] Reynolds, M. H. , Courtot, K. N. , Berkowitz, P. , Storlazzi, C. D. , Moore, J. , & Flint, E. (2015). Will the effects of sea‐level rise create ecological traps for Pacific island seabirds? PLoS ONE, 10(9), Article e0136773. 10.1371/journal.pone.0136773 PMC4580421 26398209

[cobi70335-bib-0078] Russell, J. C. , Caut, S. , Anderson, S. H. , & Lee, M. (2015). Invasive rat interactions and over‐invasion on a coral atoll. Biological Conservation, 185, 59–65. 10.1016/j.biocon.2014.10.001

[cobi70335-bib-0079] Samaniego, A. , Griffiths, R. , Gronwald, M. , Murphy, F. , Rohellec, M. L. , Oppel, S. , Meyer, J. Y. , & Russell, J. (2020). A successful pacific rat Rattus exulans eradication on tropical reiono island (Tetiaroa atoll, french polynesia) despite low baiting rates . https://researchspace.auckland.ac.nz/handle/2292/51383

[cobi70335-bib-0080] Shiels, A. B. , Pitt, W. C. , Sugihara, R. T. , & Witmer, G. W. (2014). Biology and impacts of Pacific island invasive species. 11. *Rattus rattus*, the black rat (Rodentia: Muridae). Pacific Science, 68(2), 145–184. 10.2984/68.2.1

[cobi70335-bib-0081] Soanes, L. M. , Green, J. A. , Bolton, M. , Milligan, G. , Mukhida, F. , & Halsey, L. G. (2021). Linking foraging and breeding strategies in tropical seabirds. Journal of Avian Biology, 52(7), Article e02670. 10.1111/jav.02670

[cobi70335-bib-0082] Sommerfeld, J. , Kato, A. , Ropert‐Coudert, Y. , Garthe, S. , Wilcox, C. , & Hindell, M. A. (2015). Flexible foraging behaviour in a marine predator, the Masked booby (*Sula dactylatra*), according to foraging locations and environmental conditions. Journal of Experimental Marine Biology and Ecology, 463, 79–86. 10.1016/j.jembe.2014.11.005

[cobi70335-bib-0083] Spaggiari, J. , Barré, N. , Baudat‐Franceschi, J. , Borsa, P. , & Colin, F. (2006). New Caledonian seabirds. In C. Payri & B. Richer de Forges (Eds.), Compendium of marine species from New Caledonia (pp. 371–384) Centre IRD de Bondy. http://www.documentation.ird.fr/hor/fdi:010038901

[cobi70335-bib-0084] Spatz, D. R. , Young, L. C. , Holmes, N. D. , Jones, H. P. , VanderWerf, E. A. , Lyons, D. E. , Kress, S. , Miskelly, C. M. , & Taylor, G. A. (2023). Tracking the global application of conservation translocation and social attraction to reverse seabird declines. Proceedings of the National Academy of Sciences, 120(16), Article e2214574120. 10.1073/pnas.2214574120 PMC10120044 37036988

[cobi70335-bib-0085] Steibl, S. , Kench, P. S. , Young, H. S. , Wegmann, A. S. , Holmes, N. D. , Bunbury, N. , Teavai‐Murphy, T. H. , Davies, N. , Murphy, F. , & Russell, J. C. (2024). Rethinking atoll futures: Local resilience to global challenges. Trends in Ecology & Evolution, 39(3), 258–266. 10.1016/j.tree.2023.11.004 38114338

[cobi70335-bib-0086] Steibl, S. , Steiger, S. , Wegmann, A. S. , Holmes, N. D. , Young, H. S. , Carr, P. , & Russell, J. C. (2024). Atolls are globally important sites for tropical seabirds. Nature Ecology & Evolution, 8(10), 1907–1915. 10.1038/s41559-024-02496-4 39147843

[cobi70335-bib-0087] Stoddart, D. R. , & Steers, J. A. (1977). The nature and origin of coral reef islands. In O. A. Jones & R. Endean (Eds.), Biology and geology of coral reefs (pp. 59–105). Academic Press. 10.1016/B978-0-12-395528-9.50011-7

[cobi70335-bib-0088] Surman, C. A. , & Wooller, R. D. (2003). Comparative foraging ecology of five sympatric terns at a sub‐tropical island in the eastern Indian Ocean. Journal of Zoology, 259(3), 219–230. 10.1017/S0952836902003047

[cobi70335-bib-0089] Thibault, J.‐C. , & Bretagnolle, V. (2007). Atlas des oiseaux marins nicheurs de Polynésie Française et du groupe Pitcairn. Société d'Ornithologie de Polynésie.

[cobi70335-bib-0090] Thibault, J.‐C. , Cibois, A. , & Meyer, J.‐Y. (2015). Birds of Uvea (Wallis), Futuna and Alofi islands (South‐West Pacific): An update. Notornis, 62, 30–37.

[cobi70335-bib-0091] Thibault, M. , Weston, M. A. , Ravache, A. , & Vidal, E. (2020). Flight‐initiation response reflects short‐ and long‐term human visits to remote islets. Ibis, 162(3), 1082–1087. 10.1111/ibi.12810

[cobi70335-bib-0092] Tobias, J. A. , Sheard, C. , Pigot, A. L. , Devenish, A. J. M. , Yang, J. , Sayol, F. , Neate‐Clegg, M. H. C. , Alioravainen, N. , Weeks, T. L. , Barber, R. A. , Walkden, P. A. , MacGregor, H. E. A. , Jones, S. E. I. , Vincent, C. , Phillips, A. G. , Marples, N. M. , Montaño‐Centellas, F. A. , Leandro‐Silva, V. , Claramunt, S. , … Schleuning, M. (2022). AVONET: Morphological, ecological and geographical data for all birds. Ecology Letters, 25(3), 581–597. 10.1111/ele.13898 35199922

[cobi70335-bib-0093] VanderWerf, E. A. , Young, L. C. , Crow, S. E. , Opie, E. , Yamazaki, H. , Miller, C. J. , Anderson, D. G. , Brown, L. S. , Smith, D. G. , & Eijzenga, J. (2014). Increase in wedge‐tailed shearwaters and changes in soil nutrients following removal of alien mammalian predators and nitrogen‐fixing plants at Kaena Point, Hawaii. Restoration Ecology, 22(5), 676–684. 10.1111/rec.12126

[cobi70335-bib-0094] Wagenmakers, E.‐J. , & Farrell, S. (2004). AIC model selection using Akaike weights. Psychonomic Bulletin & Review, 11(1), 192–196. 10.3758/BF03206482 15117008

[cobi70335-bib-0095] Weimerskirch, H. , Corre, M. , Bost, C. , Ballance, L. , & Pitman, R. (2009). L'avifaune de l’île Clipperton et l’écologie des oiseaux marins. In Clipperton, environnement et biodiversité d'un microcosme océanique (pp. 381–392). Publications scientifiques du Muséum National d'Histoire Naturelle‐IRD Éditions.

[cobi70335-bib-0096] Weimerskirch, H. , Corre, M. L. , Kai, E. T. , & Marsac, F. (2010). Foraging movements of great frigatebirds from Aldabra Island: Relationship with environmental variables and interactions with fisheries. Progress in Oceanography, 86(1), 204–213. 10.1016/j.pocean.2010.04.003

[cobi70335-bib-0097] Weimerskirch, H. , de Grissac, S. , Ravache, A. , Prudor, A. , Corbeau, A. , Congdon, B. , McDuie, F. , Bourgeois, K. , Dromzée, S. , Butscher, J. , Menkes, C. , Allain, V. , Vidal, E. , Jaeger, A. , & Borsa, P. (2020). At‐sea movements of wedge‐tailed shearwaters during and outside the breeding season from four colonies in New Caledonia. Marine Ecology Progress Series, 633, 225–238. 10.3354/meps13171

[cobi70335-bib-0098] Weimerskirch, H. , Le Corre, M. , Jaquemet, S. , Potier, M. , & Marsac, F. (2004). Foraging strategy of a top predator in tropical waters: Great frigatebirds in the Mozambique Channel. Marine Ecology Progress Series, 275, 297–308. 10.3354/meps275297

[cobi70335-bib-0099] Weimerskirch, H. , Le Corre, M. , Ropert‐Coudert, Y. , Kato, A. , & Marsac, F. (2005). The three‐dimensional flight of red‐footed boobies: Adaptations to foraging in a tropical environment? Proceedings of the Royal Society B: Biological Sciences, 272(1558), 53–61. 10.1098/rspb.2004.2918 PMC1634943 15875570

[cobi70335-bib-0100] Woodroffe, C. D. (2008). Reef‐island topography and the vulnerability of atolls to sea‐level rise. Global and Planetary Change, 62(1), 77–96. 10.1016/j.gloplacha.2007.11.001

