# Supplementary material for: Assessing responses of reef island seabirds to environmental and anthropogenic drivers
Source: Conserv Biol. 2026 Jul 9;40(5):e70335. doi: 10.1111/cobi.70335 (PMC13613561; doi:10.1111/cobi.70335)
Supplement: Supplementary file 1 [file COBI-40-e70335-s001.docx]

Supporting Information - ***Contrasted responses of reef island seabirds to environmental and anthropogenic drivers call for complementary conservation strategies***

Appendix S1: List of study sites (adapted from Berr, Millon et al., 2023). Sites hosting active rodent populations at the time of the study are highlighted in bold. Sites from the Chesterfield and d’Entrecasteaux subregions are considered Remote CRIS, while sites from the South-western and northern lagoon are Mainland CRIS.

| Subregion | Site | X | Y | Surface (m²) | Source | Date |
| --- | --- | --- | --- | --- | --- | --- |
| Chesterfield | Avon Nord | 158.250042 | -19.511133 | 33758 | ESRI | 2021 |
|  | Avon Sud | 158.237024 | -19.531058 | 25338 | ESRI | 2021 |
|  | Bampton | 158.603516 | -19.121937 | 42847 | Google Earth | 2019 |
|  | Caye du Nord-Est | 158.462376 | -19.868589 | 1659 | Google Earth | 2019 |
|  | Caye du Sud-Est | 158.479246 | -19.910117 | 111 | Google Earth | 2019 |
|  | Caye n°1 | 158.384115 | -19.912565 | 337 | Google Earth | 2019 |
|  | Caye n°10 | 158.457186 | -19.961416 | 122 | Google Earth | 2019 |
|  | Caye n°2 | 158.39529 | -19.918696 | 1340 | Google Earth | 2019 |
|  | Caye n°3 | 158.397892 | -19.920853 | 7389 | Google Earth | 2019 |
|  | Caye n°4 | 158.407751 | -19.925549 | 3692 | Google Earth | 2019 |
|  | Caye n°5 | 158.411279 | -19.929018 | 1102 | Google Earth | 2019 |
|  | Caye n°6 | 158.419093 | -19.931776 | 1250 | Google Earth | 2019 |
|  | Caye n°7 | 158.423997 | -19.934606 | 3820 | Google Earth | 2019 |
|  | Caye n°8 | 158.431084 | -19.939984 | 85 | Google Earth | 2019 |
|  | Caye n°9 | 158.440756 | -19.947374 | 185 | Google Earth | 2019 |
|  | Caye Ouest Bampton | 158.560325 | -19.122519 | 2941 | ESRI | 2021 |
|  | Caye Sud Île Longue | 158.326348 | -19.884134 | 7031 | Google Earth | 2019 |
|  | Caye Sud Reynard | 158.882397 | -19.440092 | 4154 | ESRI | 2021 |
|  | Îlot du Mouillage 1 | 158.470046 | -19.88364 | 22786 | Berr et al. 2023 | 2022 |
|  | Îlot du Mouillage 2 | 158.472396 | -19.889031 | 17562 | Berr et al. 2023 | 2022 |
|  | Îlot du Mouillage 3 | 158.474715 | -19.894753 | 18543 | Berr et al. 2023 | 2022 |
|  | Îlot du Mouillage 4 | 158.476682 | -19.900068 | 5314 | Berr et al. 2023 | 2022 |
|  | Îlot du Nord-Est | 158.459015 | -19.859901 | 16892 | Berr et al. 2023 | 2022 |
|  | Îlot du Passage | 158.380382 | -19.910874 | 74468 | Google Earth | 2019 |
|  | Îlot du Sud-Est | 158.478413 | -19.906926 | 12593 | Berr et al. 2023 | 2022 |
|  | Îlot Nord du Passage | 158.35919 | -19.90262 | 26163 | Berr et al. 2023 | 2022 |
|  | Îlot Sud Île Longue | 158.33616 | -19.891211 | 20560 | Berr et al. 2023 | 2022 |
|  | **Longue^1^** | **158.310881** | **-19.869295** | **246694** | **Berr et al. 2023** | **2022** |
|  | Loop | 158.476362 | -19.969085 | 59337 | Berr et al. 2023 | 2022 |
|  | Reynard | 158.941347 | -19.214781 | 80472 | ESRI | 2021 |
|  | Tortue | 158.331462 | -19.887831 | 11173 | Berr et al. 2023 | 2022 |
|  | Totof | 158.467302 | -19.878156 | 2085 | Google Earth | 2019 |
| Entrecasteaux | Fabre | 163.009809 | -18.294809 | 133709 | ESRI | 2022 |
|  | Huon | 162.959106 | -18.039329 | 174686 | ESRI | 2021 |
|  | Le Leizour | 163.042552 | -18.284372 | 180759 | ESRI | 2022 |
|  | Surprise | 163.086052 | -18.481155 | 198666 | ESRI | 2021 |
| South-western lagoon | Amédée | 166.468141 | -22.477448 | 54820 | ESRI | 2021 |
|  | Gi | 166.851254 | -22.722562 | 71840 | Berr et al. 2023 | 2022 |
|  | Iéroué | 166.827052 | -22.603514 | 53485 | Berr et al. 2023 | 2022 |
|  | Koko | 166.932974 | -22.880821 | 19272 | Berr et al. 2023 | 2022 |
|  | Kouaré | 166.801459 | -22.776876 | 54702 | Berr et al. 2023 | 2022 |
|  | M'Bé | 166.561509 | -22.480926 | 11105 | ESRI | 2021 |
|  | M'Boré | 166.916344 | -22.802889 | 9464 | Berr et al. 2023 | 2022 |
|  | N'Da | 166.877939 | -22.846558 | 51124 | Berr et al. 2023 | 2022 |
|  | N'Di | 166.851155 | -22.579629 | 775 | ESRI | 2022 |
|  | N'Do | 166.968681 | -22.683851 | 51533 | Berr et al. 2023 | 2022 |
|  | N'Gé | 166.850645 | -22.693315 | 49274 | Berr et al. 2023 | 2022 |
|  | Noé | 166.822216 | -22.539441 | 37894 | Berr et al. 2023 | 2022 |
|  | Petit Koko | 166.943937 | -22.858344 | 2034 | Berr et al. 2023 | 2022 |
|  | Puemba | 166.829886 | -22.532206 | 72173 | Berr et al. 2023 | 2022 |
|  | Pûmbo | 166.835734 | -22.520614 | 4143 | Berr et al. 2023 | 2022 |
|  | Rédika | 166.611158 | -22.515772 | 100322 | Berr et al. 2023 | 2022 |
|  | Téré | 166.847943 | -22.788756 | 19788 | Berr et al. 2023 | 2022 |
|  | Totéa | 166.973669 | -22.720437 | 15433 | Berr et al. 2023 | 2022 |
|  | Ua | 166.811835 | -22.708326 | 52940 | Berr et al. 2023 | 2022 |
|  | Uatérembi | 166.811411 | -22.678919 | 71566 | Berr et al. 2023 | 2022 |
|  | Uatio | 166.795599 | -22.710445 | 57589 | Berr et al. 2023 | 2022 |
|  | Uié | 166.917203 | -22.722679 | 26329 | Berr et al. 2023 | 2022 |
|  | Vua | 166.747085 | -22.576871 | 63010 | Berr et al. 2023 | 2022 |
|  | Contrariété | 165.05548 | -21.466188 | 7158 | Berr et al. 2023 | 2020 |
|  | **Goldfield^2^** | **166.150515** | **-22.141476** | **33192** | **Berr et al. 2023** | **2020** |
|  | Île aux Canards | 166.435492 | -22.312472 | 8944 | Berr et al. 2023 | 2020 |
|  | Konduyo | 165.781237 | -21.868302 | 22632 | Berr et al. 2023 | 2020 |
|  | **Lange^1^** | **166.262045** | **-22.17735** | **41857** | **Géorep.nc** | **2011** |
|  | **Larégnère^1^** | **166.319626** | **-22.327343** | **10861** | **Géorep.nc** | **2011** |
|  | **Maître^4^** | **166.409681** | **-22.334231** | **67994** | **Géorep.nc** | **2011** |
|  | M'Ba | 166.202297 | -22.216814 | 318811 | ESRI | 2021 |
|  | M'Bé Kouen | 166.222169 | -22.264827 | 2224 | Berr et al. 2023 | 2020 |
|  | M'Bo | 166.225838 | -22.243053 | 149452 | Géorep.nc | 2011 |
|  | N'Digoro | 165.733198 | -21.863038 | 1614 | Berr et al. 2023 | 2020 |
|  | Pandanus | 166.258815 | -22.203222 | 1122 | ESRI | 2022 |
|  | Petit Ténia | 166.026329 | -22.062161 | 43790 | Berr et al. 2023 | 2020 |
|  | **Ronde^4^** | **166.240202** | **-22.197788** | **44289** | **Berr et al. 2023** | **2020** |
|  | **Signal^4^** | **166.293013** | **-22.296504** | **121471** | **Berr et al. 2023** | **2020** |
|  | **Verte^4^** | **165.458964** | **-21.658797** | **95042** | **Berr et al. 2023** | **2020** |
| Northen lagoon | Bayes | 165.480619 | -20.901732 | 10194 | Berr et al. 2023 | 2020 |
|  | Boisé | 165.491019 | -20.91193 | 2448 | Berr et al. 2023 | 2020 |
|  | **Camille^3^** | **165.147689** | **-20.715676** | **101044** | **Berr et al. 2023** | **2020** |
|  | **Hienga^3^** | **165.051788** | **-20.663244** | **102898** | **Berr et al. 2023** | **2020** |
|  | **Hiengabat^3^** | **164.987033** | **-20.645466** | **169305** | **Berr et al. 2023** | **2020** |
|  | **Hienghène^3^** | **164.937693** | **-20.624394** | **212374** | **Géorep.nc** | **2012** |
|  | **Hiengu^3^** | **165.090674** | **-20.688775** | **67318** | **Géorep.nc** | **2012** |
|  | Sable | 165.271308 | -20.745621 | 969 | Berr et al. 2023 | 2020 |
|  | Solitaire | 165.456636 | -20.880985 | 1103 | Berr et al. 2023 | 2020 |
|  | Thigit | 164.917806 | -20.625402 | 48264 | Berr et al. 2023 | 2021 |
|  | Tibarama | 165.371317 | -20.920492 | 25818 | Géorep.nc | 2020 |
|  | Carrey | 163.954402 | -20.382998 | 6129 | Géorep.nc | 2020 |
|  | Cayao | 164.315896 | -20.725041 | 5544 | Berr et al. 2023 | 2022 |
|  | **Deverd^1,2^** | **164.325448** | **-20.767674** | **33177** | **Géorep.nc** | **2020** |
|  | Double | 164.128567 | -20.467653 | 50836 | Géorep.nc | 2020 |
|  | Goenghu | 164.114787 | -20.393307 | 31184 | Géorep.nc | 2020 |
|  | Kendec | 164.257012 | -20.667341 | 9164 | Géorep.nc | 2020 |
|  | Magonne | 164.206951 | -20.55447 | 5781 | Géorep.nc | 2020 |
|  | Nana | 164.126031 | -20.427419 | 17337 | Géorep.nc | 2020 |
|  | Ouanne | 164.025269 | -20.346622 | 23515 | Berr et al. 2023 | 2021 |
|  | Pouh | 164.118783 | -20.510048 | 11593 | Géorep.nc | 2020 |
|  | Tangadiou | 164.223907 | -20.551974 | 58839 | Berr et al. 2023 | 2021 |
|  | Ti-Ac | 164.083398 | -20.367463 | 101362 | Géorep.nc | 2020 |
|  | Tiam'Bouène | 164.036324 | -20.405598 | 152007 | Géorep.nc | 2020 |
|  | Yan'Dagouet | 164.078527 | -20.456428 | 36273 | Berr et al. 2023 | 2021 |

^1^: presence of house mouse *Mus musculus*; ^2^: presence of Polynesian rat *Rattus exulans*; ^3^: presence of black rat *Rattus rattus*; ^4^ presence of undetermined rodent species

| Order | Family | Name | Common name | Red List status | Number of breeding sites | Combined population size on study sites (bp) | % of population breeding on mainland CRIS |
| --- | --- | --- | --- | --- | --- | --- | --- |
| Charadriiformes | *Laridae* | *Anous minutus* | Black noddy | LC | **25** | **71 339** | **51.7** |
|  |  | *Anous stolidus* | Brown noddy | LC | **38** | **12 508** | **11.1** |
|  |  | *Larus novaehollandiae* | Silver gull | LC | **34** | **255** | **100** |
|  |  | *Onychoprion anaethetus* | Bridled tern | LC | **27** | **707** | **97.2** |
|  |  | *Onychoprion fuscatus* | Sooty tern | LC | **15** | **139 116** | **<0.1** |
|  |  | *Sterna dougallii* | Roseate tern | LC | **34** | **4 637** | **99.9** |
|  |  | *Sterna sumatrana* | Black-naped tern | LC | **43** | **772** | **67.9** |
|  |  | *Sternula nereis* | Fairy tern | VU | **21** | **161** | **82.6** |
|  |  | *Thalasseus bergii* | Greater-crested tern | LC | **29** | **1 674** | **82.0** |
| Procellariiformes | *Procellariidae* | *Ardenna pacifica* | Wedge-tailed shearwater | LC | **59** | **254 681** | **87.1** |
| Suliformes | *Fregatidae* | *Fregata ariel* | Lesser frigatebird | LC | **10** | **3 516** | **0** |
|  |  | *Fregata minor* | Great frigatebird | LC | **14** | **1 369** | **0** |
|  | *Sulidae* | *Sula dactylatra* | Masked booby | LC | **22** | **572** | **0.3** |
|  |  | *Sula leucogaster* | Brown booby | LC | **23** | **4 309** | **1.7** |
|  |  | *Sula sula* | Red-footed booby | LC | **19** | **12 510** | **1.2** |

Appendix S2: Combined population sizes of seabird species across all study sites (100 CRIs). Adapted from Berr, Millon et al., 2023.

*Appendix S3: Data sources for seabird census data and information on invasive rodents*

| Area | Survey period | Source |
| --- | --- | --- |
| Northern lagoon | 2005-2006 | Baudat-Franceschi, 2006 |
|  | 2016-2020 | Provincial rangers, *unpublished data* |
|  | 2018-2020 | *this study* |
| South-western lagoon | 2009-2011 | Baudat-Franceschi, 2012 |
|  | 2015-2022 | Provincial rangers, *unpublished data* |
|  | 2018-2023 | *this study* |
| Chesterfield-Bampton | 2001-2009 | Borsa et al., 2010 |
|  | 2010 | Baudat-Franceschi, 2011 |
|  | 2017-2022 | *this study* |
| d'Entrecasteaux reefs | 2003-2008 | Philippe-Lesaffre et al., 2023 |
|  | 2007-2015 | Yearly reports, Ornithological Society of New Caledonia^1^ |
|  | 2017-2021 | *this study* |

*^1^Compiled in Borsa et al., 2021*

| Species | 1-Beach/bare substrate | 2-Savanna | 3-Mixed shrub^1^ | 4-Bay cedar | 5-Other |
| --- | --- | --- | --- | --- | --- |
| Black noddy |  |  | X |  |  |
| Brown noddy | X | X |  | X |  |
| Silver gull |  |  | X | X | X |
| Bridled tern |  |  | X | X | X |
| Sooty tern | X | X |  |  |  |
| Roseate tern | X |  |  |  |  |
| Black-naped tern | X |  |  |  |  |
| Fairy tern | X |  |  |  |  |
| Greater-crested tern | X | X |  |  |  |
| Wedge-tailed shearwater |  | X | X | X | X |
| Lesser frigatebird |  | X |  |  |  |
| Great frigatebird |  |  | X |  |  |
| Masked booby | X | X |  |  |  |
| Brown booby | X | X |  |  |  |
| Red-footed booby |  |  | X |  |  |

*Appendix S4: Habitat use per species. X indicate possible breeding by the corresponding species.*

*^1^Either Pisonia grandis or octopus bush tree Heliotropium foertherianum*

*Appendix S5: Pearson’s correlation coefficient measured between PCA (Geography) or MFA (Human visitation) axes and original quantitative variables. Red indicates R > |0.7| and black indicates R > |0.5|. The two boxplots in the lower panel display MFA coordinates (Visitation) per category of tourism-related land use (top) and type of terrestrial reserve (bottom).*

*Appendix S6: List of 32 original geography variables (A) and 7 original human variables (B) prior to principal component (A) or multifactorial (B) analysis. (C) lists additional drivers that were retained without transformation. Adapted from Berr, Millon et al., 2023.*

|  | Name | Description | Units | Mean | Range |
| --- | --- | --- | --- | --- | --- |
| (A)^1^ | Reef_buffer (8 variables) | Proportion (1-0) of each category in eight buffers drawn from CRI shoreline (0.5, 1, 5, 10, 50, 100, 150, 200 km) | - | - | - |
|  | Lagoon_buffer (8 variables) |  | - | - | - |
|  | Land_buffer (8 variables) |  | - | - | - |
|  | Pelagic_buffer (8 variables) |  | - | - | - |
| (B) | log(Distance to Noumea) | log-transformed distance to Nouméa and to the closest harbor | (original distance in km) | 5.26 | 5.95 |
|  | log(Distance to closest harbor) |  | (original distance in km) | 4.8 | 5.95 |
|  | Nb harbors 10km | Number of harbors in 10 and 60 km buffers drawn from CRI shoreline | - | 0.5 | 16 |
|  | Nb harbors 60km |  | - | 6.33 | 25 |
|  | Source population size | Population size of urban areas from which visitation occur | number of inhabitants | 55 130 | 0-160 672 |
|  | Reserve type | Categorical description of the type of terrestrial reserve: Absent, Adaptive (access restrictions in case of breeding activity), Partial (part of the CRI permanently restricted) and Permanent (whole area restricted from access) | | | |
|  | Infrastructure / Tourism | Categorical description of tourism-related land-use: high visitation (permanent touristic infrastructure and/or possible daily taxi-boat visit), medium visitation (traces of occasional visits and camping activity), low visitation (no visible signs of human visitation) | | | |
|  | Islet shape | Polsby-Popper score of shape compactness (0-1) | - | 0.6 | 0.06-0.96 |
|  | Rodent presence | Binary categorization of rodent presence: 0-absent, 1- one among black rat *Rattus rattus*, Polynesian rat *Rattus exulans*, domestic mouse *Mus musculus*, unidentified rodent, multiple species. | | | |

^1^Source of reference geographical data: Géorep.nc

Appendix S7: Measured values of the Visitation predictor (approximation of human disturbance) for the 100 study sites.

|  | Southwestern lagoon | Northern lagoon | Entrecasteaux |
| --- | --- | --- | --- |
| Chesterfield | 0.001 | **0.001** | **0.903** |
| Entrecasteaux | 0.032 | **0.393** |  |
| Northern Lagoon | 0.008 |  |  |

Appendix S8: ANalysis Of SIMilarities (ANOSIM) of breeding species assemblages performed between all pairs of subregions. A p-value < 0.05 indicates a significant difference between the considered groups.

| Species | Intercept | Breeding habitat | Marine.1 | Marine.2 | Islet shape | Human visitation | Rodent presence | \|Δw\| |
| --- | --- | --- | --- | --- | --- | --- | --- | --- |
| Black noddy | **-3.28***[-4.62;-1.94]** | **0.43***[0.24;0.61]** |  | 0.7[-0.23;1.62] | -0.23[-1.03;0.56] | -1.07°[-2.01;-0.13] | 1.25[-1.99;4.49] | 0.07 |
|  | **-3.24***[-4.57;-1.91]** | **0.42***[0.24;0.59]** | 1.00°[0.14;1.87] | 0.41[-0.43;1.26] | -0.24[-1.03;0.55] |  | 1.26[-1.81;4.34] |  |
| Brown noddy | **-8.27*[-13.46;-3.08]** | **0.83*[0.26;1.40]** |  | **1.87***[0.86;2.88]** | 0.31[-0.60;1.22] | **-2.31***[-3.41;-1.20]** | -1.81[-6.77;3.15] | 0.85 |
| Silver gull | **-5.51*[-9.53;-1.50]** | **0.43*[0.06;0.79]** | **-2.52*[-4.24;-0.79]** | **1.52[0.69;-2.35]** | -1.05[-2.11;0.02] |  | 0.24[-1.45;1.93] | 0.99 |
| Bridled tern | -2.10°[-3.86;-0.35] | 0.15[-0.06;0.35] | -0.83[-1.62;-0.04] | **1.57***[0.88;2.26]** | 0.06[-0.70;0.83] |  | -1.27[-3.59;1.05] | 0.48 |
| Sooty tern | -4.96[-9.98;0.07] | 0.44[-0.08;0.95] | **1.40*[0.41;2.38]** | 0.78°[-0.14;1.70] | 0.6[-0.13;1.34] |  | -15.94[-3169.09;3137.22] | 0.18 |
| Roseate tern | 2.5[-4.32;9.32] | -0.67[-1.39;0.05] | **-1.80**[-2.74;-0.86]** | **1.37***[0.69;2.04]** | -0.15[-0.94;0.64] |  | -2.32[-4.60;-0.05] | 0.55 |
| Black-naped tern | -0.66[-3.43;2.10] | 0.2[-0.30;0.70] |  | **0.66*[0.14;1.18]** | 0.15[-0.37;0.67] | 0.55[0.02;1.09] | -1.84[-3.69;0.01] | 0.08 |
|  | -0.52[-2.97;1.93] | 0.15[-0.34;0.64] | -0.54°[-1.03;-0.04] | **0.77**[0.28;1.26]** | 0.12[-0.42;0.66] |  | -1.68[-3.41;0.06] |  |
| Fairy tern | -1.23[-3.85;1.40] | -0.1[-0.70;0.49] | **-1.01*[-1.80;-0.22]** | 0.12[-0.44;0.68] | -0.53[-1.21;0.14] |  | -1.24[-2.95;0.46] | 0.85 |
| Greater crested tern | -1.11[-2.80;0.58] | 0.01[-0.35;0.37] |  | **0.92***[0.40;1.44]** | 0.29[-0.25;0.83] | 0.06[-0.46;0.58] | 0.34[-1.21;1.88] | 0.06 |
|  | -1.12[-2.84;0.60] | 0.02[-0.35;0.39] | -0.2[-0.73;0.33] | **0.93***[0.41;1.44]** | 0.28[-0.28;0.83] |  | 0.29[-1.25;1.84] |  |
| Wedge-tailed shearwater | **-5.53**[-8.94;-2.11]** | **0.67**[0.31;1.02]** |  | **0.66*[0.01;1.30]** | -0.37[-1.05;0.32] | 0.65°[-0.03;1.33] | 0.15[-1.88;2.18] | 0.03 |
|  | **-5.53**[-8.94;-2.13]** | **0.67**[0.31;1.02]** | -0.59[-1.22;0.03] | **0.78*[0.12;1.43]** | -0.39[-1.10;0.31] |  | 0.27[-1.68;2.22] |  |
| Lesser frigatebird | **-8.31*[-14.71;-1.91]** | 0.61[-0.10;1.32] | 1.64°[0.21;3.06] | 0.03[-1.12;1.18] | 0.08[-0.70;0.87] |  | 0.76[-3.25;4.76] | NA |
| Great frigatebird | **-9.71**[-15.28;-4.14]** | **0.89*[0.31;1.47]** | **3.54*[1.30;5.78]** | -1.08[-2.83;0.67] | -0.97[-2.37;0.43] |  | 0.07[-22.00;22.13] | NA |
| Masked booby | **-14.21**[-22.14;-6.28]** | **1.29*[0.44;2.13]** |  | 0.78[-0.57;2.13] | -0.43[-1.40;0.54] | **-2.53**[-3.85;-1.21]** | -1.88[-8.60;4.84] | 0.7 |
| Brown booby | **-20.30**[-32.02;-8.58]** | **1.96*[0.71;3.22]** |  | 0.63[-0.77;2.02] | 0.3[-0.91;1.51] | **-3.01**[-4.57;-1.46]** | -3.28[-13.31;6.75] | 0.91 |
| Red-footed booby | **-10.44*[-17.57;-3.32]** | **1.28*[0.32;2.24]** |  | 0.95[-0.67;2.58] | -1.36[-3.68;0.95] | **-5.39*[-9.58;-1.20]** | -4.47[-62.91;53.98] | 0.75 |

Appendix S9: Coefficient estimates for the site selection models (binomial generalized linear model with logit link), considering all 100 study sites. °: Benjamini-Hochberg adjusted p-value < 0.1; *<0.05; **<0.01; ***<0.001. Significant effects are highlighted in bold. Human visitation and Marine.1 (proxy of site isolation from the mainland) were correlated and therefore mutually exclusive: for each species, only the predictor with the highest Akaike weight among the two was retained. When the two predictors exhibited small differences in weight (|Δw| < 0.1), both versions of the models were displayed.

*Appendix S10: Coefficient estimates for the species abundance models (generalized linear model with a Poisson link). °: Benjamini-Hochberg adjusted p-value < 0.1; *<0.05; **<0.01; ***<0.001. Significant effects are highlighted in bold.* *Only sites hosting breeding populations of each species were included, and rodent presence was only considered when at least three sites hosted both rodents and the corresponding species. ^1^the visitation predictor was not included for six species only or mostly breeding on remote CRI, that receive very limited visitation.*

| Species | Intercept | Breeding habitat area | Habitat:Visitation | Habitat:Rodent presence | AICc |
| --- | --- | --- | --- | --- | --- |
| Wedge-tailed shearwater | **-2.11***[-2.17;-2.06]** | **0.99***[ 0.99; 1.00]** | **-0.09***[-0.09;-0.09]** |  | 128407.7515 |
|  | **-1.87***[-1.93;-1.82]** | **0.95***[ 0.94; 0.95]** |  | **-0.07***[-0.07;-0.07]** | 163171.6516 |
| Red-footed booby | **-1.18***[-1.33;-1.03]** | **0.83***[ 0.81; 0.84]** | *^1^* |  |  |
| Brown noddy | **-2.02***[-2.19;-1.85]** | **0.73***[ 0.72; 0.75]** | **0.06***[ 0.06; 0.07]** |  |  |
| Black noddy | **2.05***[ 1.99; 2.12]** | **0.67***[ 0.66; 0.67]** | **-0.06***[-0.07;-0.06]** |  |  |
| Great frigatebird | **-1.61***[-2.03;-1.20]** | **0.66***[ 0.62; 0.70]** | *^1^* |  |  |
| Brown booby | **-1.78***[-2.08;-1.48]** | **0.66***[ 0.64; 0.69]** | *^1^* |  |  |
| Masked booby | **-3.25***[-4.15;-2.36]** | **0.62***[ 0.54; 0.71]** | *^1^* |  |  |
| Sooty tern | **6.24***[ 6.19; 6.28]** | **0.29***[ 0.29; 0.30]** | *^1^* |  |  |
| Bridled tern | **1.50***[ 0.95; 2.04]** | **0.17***[ 0.12; 0.22]** | **0.02*[ 0.00; 0.03]** |  |  |
| Greater crested tern | **3.04***[ 2.71; 3.37]** | **0.12***[ 0.08; 0.16]** | -0.01[-0.02; 0.01] |  |  |
| Black-naped tern | **1.91***[ 1.26; 2.56]** | **0.11**[ 0.03; 0.19]** | **0.03**[ 0.01; 0.04]** |  |  |
| Fairy tern | 1.28[-0.50; 2.88] | 0.10[-0.10; 0.32] | 0.03[-0.01; 0.07] |  |  |
| Roseate tern | **6.06***[ 5.77; 6.35]** | **-0.17***[-0.21;-0.14]** | **0.05***[ 0.04; 0.06]** |  |  |
| Silver gull | **4.98***[ 3.86; 6.06]** | **-0.25***[-0.36;-0.14]** | **-0.06***[-0.09;-0.03]** |  | 286.2008 |
|  | **4.67***[ 3.51; 5.80]** | **-0.25***[-0.37;-0.13]** |  | 0.01[-0.03; 0.05] | 306.3865 |
| Lesser frigatebird | **8.90***[ 8.53; 9.27]** | **-0.30***[-0.34;-0.26]** | *^1^* |  |  |

Appendix S11: Species-wise comparison of Akaike weights for the correlated (Pearson’s R >0.7) Marine.1 (approximation of site isolation from the mainland) and Human visitation predictors. Species are sorted by increasing weight of the Marine.1 predictor (top to bottom).

Appendix S12: Differences in breeding densities of wedge-tailed shearwaters in the absence or presence of invasive rodent species.
